# Testing Trait-State Isomorphism in a New Domain: An Exploratory Manipulation of Openness to Experience

**DOI:** 10.3389/fpsyg.2018.01964

**Published:** 2018-10-15

**Authors:** Zachary M. van Allen, John M. Zelenski

**Affiliations:** Department of Psychology, Carleton University, Ottawa, ON, Canada

**Keywords:** openness to experience, trait-state isomorphism, big five, creativity, personal growth

## Abstract

The trait-state isomorphism hypothesis holds that personality traits and states (i.e., trait-related behavior) are characterized by similar outcomes (Fleeson, 2001). Openness is associated with creative thinking, personal growth, and positive affect. Engaging in behavior associated with openness has also been found to covary with feelings of authenticity. In the present experiment, participants (*N* = 210) completed a pre-test assessment, five daily exercises designed to either be inert (control condition) or engage the behaviors and cognitions associated with openness (experimental condition), a post-test assessment, and a 2 week follow up assessment. Results supported the isomorphism hypothesis for positive affect but not creative thinking ability or personal growth. Furthermore, open behavior was only associated with authenticity for individuals high on trait openness.

## Introduction

Trait openness can be characterized as an inclination toward introspection, curiosity, aesthetic appreciation, affective sensitivity, and the exploration of ideas (Goldberg, 1990; Costa and McCrae, 1992; Connelly et al., 2014), and is associated with many desirable outcomes and processes. For example, open individuals are inclined toward personal growth (Schmutte and Ryff, 1997), demonstrate adaptive reactions toward stress (Williams et al., 2009), and score higher on tests of creative thinking ability (Feist, 1998; Carson et al., 2003; Silvia et al., 2009). Additionally, the trait of openness is associated with creative achievement in the arts and sciences (Feist, 1998), the enjoyment of mind-wandering (Wilson et al., 2014), and the increased propensity to experience aesthetic chills (McCrae, 2007) and awe (Shiota et al., 2006). Experience sampling studies have also found that the behaviors associated with openness are accompanied by elevated feelings of authenticity regardless of one’s individual disposition (Fleeson and Wilt, 2010).

Given the many benefits associated with openness, an intriguing possibility is that these outcomes could be cultivated by mimicking the behaviors associated with openness. This proposition is supported by a parallel program research into “enacted extraversion.” Trait extraversion is robustly associated with positive affect, and multiple laboratory experiments have demonstrated that individuals experience increases in positive affect when they behave in an extraverted manner (Fleeson et al., 2002; Zelenski et al., 2012, 2013), and this effect does not appear to depend on dispositional levels of extraversion (Zelenski et al., 2013). This research is grounded in the density distribution model of personality and the idea that traits and states are at least somewhat isomorphic (Fleeson, 2001). That is, personality states are similar to traits in affective and behavioral content but are manifested in momentary experience, such as over the course of minutes or hours instead of years (Fleeson, 2001).

Based on the findings of these laboratory based “enacted extraversion” experiments, some have suggested that it may be possible to manipulate “open behaviors” in a similar manner to elicit the positive cognitive benefits associated with openness (e.g., Smillie, 2013; Blackie et al., 2014; Forgeard and Eichner, 2014). For example, researchers have cited the possibility of manipulating openness to facilitate outcomes such as creativity (Forgeard and Eichner, 2014) and post-traumatic growth (Blackie et al., 2014). However, to date, this approach of “acting well to be well” has yet to be applied to openness.

Relative to the other big five factors of extraversion, agreeableness, neuroticism, and conscientiousness, the composition of traits thought to encompass the fifth factor has been subject to much debate. For example, some taxonomies classify introspection as a defining feature (e.g., Goldberg, 1999; Connelly et al., 2014) while others do not (e.g., Costa and McCrae, 1992; Ashton and Lee, 2001). In the NEO PI-R, a widely used measurement of the big five, “openness to experience^1^” is comprised of six facets: ideas (preference for intellectual interests), aesthetics (appreciation for beauty and art), fantasy (possessing an active imagination), actions (preference for novelty and variety), feelings (emotional depth and sensitivity) and values (socio-politically progressive ideals). According to this classification system, “open” individuals are generally “imaginative, sensitive to art and beauty, emotionally differentiated, behaviorally flexible, intellectually curious, and liberal in values. Closed people are down-to-earth, uninterested in art, shallow in affect, set in their ways, lacking curiosity, and traditional in values” (McCrae and Sutin, 2007; p. 258).

Openness to experience is a personality dimension primarily associated with cognitive traits (Zillig et al., 2002). For example, need for cognition (Cacioppo and Petty, 1982) is a psychological construct that refers to individual differences in the tendency to engage in and enjoy thinking and is highly correlated with openness to experience, especially the “ideas” facet (*r* = 0.78; Berzonsky and Sullivan, 1992). The open individuals’ need for cognition also manifests itself in a high tolerance for ambiguity and a low need for cognitive closure. Need for closure is a construct that measures the desire for predictability, order, and structure as well as discomfort with ambiguity (Webster and Kruglanski, 1994) and is strongly and inversely related to openness (*r* = −0.50; Onraet et al., 2011).

Given the tendency for individuals high in openness to seek out and enjoy cognitive stimulation, it is not surprising that open individuals are also more likely to become immersed in thought. The construct of absorption (Glisky et al., 1991), as assessed by the multidimensional personality questionnaire (Tellegen, 1982, Unpublished), refers to the tendency to become immersed in ones thoughts and imagination, and is highly intercorrelated with openness to experience. The tendency for open individuals to become immersed in thought may underlie the positive relationship between openness and the frequency and enjoyment of daydreaming (Wilson et al., 2014).

Openness to experience is strongly associated with self-reported creative achievement (King et al., 1996; Silvia et al., 2009). At the trait level, openness to experience is equally related to creative achievement in both the arts and sciences (Feist, 1998); however, recent research employing the big five aspects scale, which partitions openness into the traits of “openness” and “intellect,” found that openness predicts creative achievement in the arts and intellect predicts achievement in the sciences (Kaufman et al., 2015). The cognitive process most often associated with creative ability is divergent thinking. Divergent thinking, or the ability to produce multiple solutions to a single problem (Guilford, 1950), is assessed with tests such as alternate uses task (Christensen et al., 1960) which assesses the ability of the test-taker to describe as many possible uses for a common household object such as a brick or pen. Of the big five personality traits, openness and extraversion are the strongest predictors of divergent thinking (McCrae, 1987; King et al., 1996; Silvia et al., 2009; Sung and Choi, 2009).

Open individuals are also characterized by a greater sensitivity to emotions. For example, feelings facet of openness in the NEO-IPIP-120 accesses the extent to which participants “feel others’ emotions” and “experience [their own] emotions intensely.” This enhanced affective sensitivity is evidenced in correlations between openness subscales of the highly sensitive persons scale (Aron and Aron, 1997; Evans and Rothbart, 2008). For example, across two studies Sobocko and Zelenski (2015) observed positive correlations between openness and the highly sensitive persons scale subscales of aesthetics (measuring sensitivity to aesthetic stimuli; *r* = 0.47 and 0.51) and orienting sensitivity/openness (measuring awareness of internal and external sensory events; *r* = 0.46 and 0.42). Thus, open individuals are more aware of and sensitive to, their emotional states.

The relationship between openness and positive emotions has been subject to increasing empirical attention. The most recent large-scale meta-analysis on personality and well-being (Steel et al., 2008) found that openness, as assessed by NEO inventory (Costa and McCrae, 1992), is significantly related to positive affect (*r* = 0.20) but not negative affect (*r* = −0.02). Consistent with this finding, Ching et al. (2014) employed an experience sampling methodology and found that openness was a significant predictor of positive affective states in daily life across five cultures (β = 0.18–0.25). However, positive affect is a broad construct, and multiple lines of research have found positive associations between openness and more specific types of positive emotions. For example, Shiota et al. (2006) investigated the associations between the big five and the dispositional positive emotions scale (DPES) and found positive correlations between openness to experience and several subscales of the DPES including love (*r* = 0.28), compassion (*r* = 0.40), amusement (*r* = 0.20), and awe (*r* = 0.49). Furthermore Letzring and Adamcik (2015) found that openness was a significant predictor of several positively valenced items on the positive and negative affect schedule (PANAS; Watson et al., 1988) including: “inspired” (β = 0.30), “determined” (β = 0.16), and “interested” (β = 0.16). Similarly Mitte and Kampfe (2008) also found openness to be strongly associated with the positive affective state of interest. Finally, McCrae (2007) has argued that aesthetic chills, or the “experience of chills or goosebumps in response to aesthetic stimulation” (McCrae, 2007, p. 5), is the best universal marker of openness.

Trait openness has been linked with various indicators of well-being including subjective assessments of happiness (Steel et al., 2008), and authenticity (Sheldon et al., 1997). Authenticity, or the evaluation that one’s behavior is in concordance with one’s “true self,” has also been found to covary with the behaviors associated with openness. For example, across three experiments employing an experiencing sampling methodology Fleeson and Wilt (2010) found that behavior associated with extraversion, openness, agreeableness, conscientiousness, and emotional stability was consistently related to feelings of authenticity, regardless of individual disposition. This same pattern of results has also been reported in cross-sectional research (Sheldon et al., 1997) and may be influenced by the role of positive emotions in appraisals of authenticity (Lenton et al., 2013).

Another indicator of well-being associated with openness is personal growth. Personal growth, as measured by the psychological well-being scale (PWB; Ryff, 1989), assesses the extent to which individuals possess a sense of continued growth and development, and is consistently associated with openness and extraversion (Schmutte and Ryff, 1997). Schmutte and Ryff (1997) have suggested that individuals with this combination of traits have the inclination (via openness) and the energy (via extraversion) to pursue growth opportunities.

An additional openness facet associated with well-being outcomes is curiosity. Trait curiosity, or the tendency to seek out novel, complex, and challenging stimuli, has been found to correlated positively with openness (*r* = 0.39; Kashdan and Steger, 2007). The strong conceptual overlap between curiosity and openness is evidenced by the fact that curiosity is sometimes, but not always, included as a facet of openness and that both curiosity and openness are characterized by an exploratory tendency toward novel and complex stimuli. Interestingly, it has been proposed that regulating curiosity may be a means to facilitate creativity (Kashdan and Fincham, 2004); this proposition has been echoed by calls to manipulate openness in general as a means of influencing creative thinking (Blackie et al., 2014).

Two notable studies have manipulated trait openness. One study found that cognitive training in an elderly population increased trait openness (Jackson et al., 2012). In another study, researchers demonstrated that long term changes in trait openness can be induced with pharmacological substances. Specifically, MacLean et al. (2011) administered psilocybin (the active ingredient in psychedelic mushrooms) to participants and found that self-reported openness was elevated immediately following the experimental session, as well as over a year later.

An alternative approach for manipulating openness is by eliciting “open” states. Personality states are similar to traits in affective and behavioral content but are manifested in momentary experience, such as over the course of minutes or hours instead of years (Fleeson, 2001). Interestingly, some traits and states appear to be associated with similar outcomes. For example, the robust association between trait extraversion and positive affect is also observed at the state level. That is, whether through experiencing sampling methods or via experimental manipulation of extraverted behavior, extraverted states are associated with positive affect regardless of individual disposition (Fleeson, 2001; Zelenski et al., 2013). Likewise, this effect is also evident with neuroticism; neuroticism at both the trait and state level is characterized by elevated levels of negative affect (McNiel and Fleeson, 2006). Taken together these findings suggest that these traits and states are at least partially characterized by similar outcomes. This phenomenon has been dubbed trait-state isomorphism (Fleeson, 2001). Although no published experiments have investigated whether openness is also isomorphic, based on the aforementioned research, manipulating open states seems a plausible method for facilitating the outcomes associated with trait openness.

**FIGURE 1:**
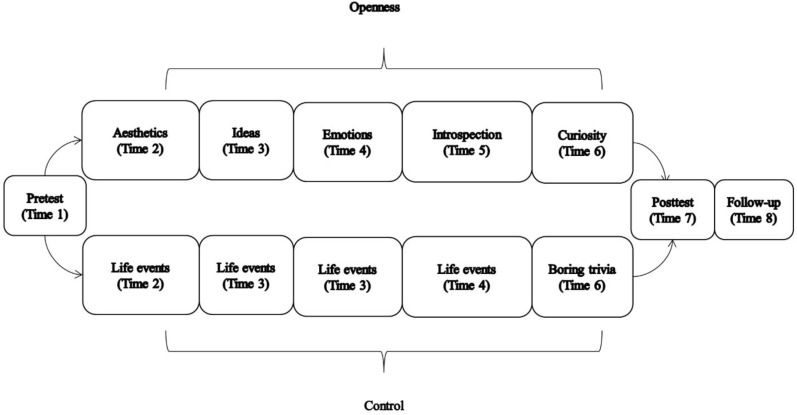
Illustration of experimental design.

In order to test whether openness is isomorphic, we designed a semi-longitudinal study consisting of eight time points spanning the course of 3 weeks. We report all data exclusions, all manipulations, and all measures used in this study. On the first day participants completed a pre-test assessment online (Time 1) that assessed personality, affect, creative thinking ability, and well-being (see **Figure 1**). For the following 5 days, participants completed one condition-specific task each day via daily online logs (Times 2–6). In the experimental condition these tasks were meant to induce open states, i.e., the behavior and cognitions associated with one of five selected facets of openness; in the control conditions participants completed a series of mundane writing tasks. In addition to the exercises, each daily log was accompanied by a brief online survey that presented participants with a test of creative thinking (alternate uses test) and self-report questionnaires assessing affect and authenticity. Following the five daily exercises, a post-test assessment (Time 7) and 2 weeks follow-up assessment (Time 8) were administered in order to capture lasting effects of the intervention, should they exist.

Given the lack of consensus regarding the defining characteristics of openness we arrived at a selection of characteristics rationally. As a starting point we began with Costa and McCrae (1992) conceptualization of openness to experience as it represents a “mainstream” view of openness. The six facets of openness to experience include: ideas, aesthetics, values, feelings, actions, and fantasy. Research clearly demonstrates the aesthetics and ideas facets to be the best markers of openness (e.g., Johnson, 1994). Additionally, evidence supports the notion that open individuals are more sensitive to and aware of their internal emotional states (e.g., Connelly et al., 2014; Sobocko and Zelenski, 2015). Therefore, the preference for ideas, the appreciation of aesthetics, and emotional awareness were retained as defining characteristic of openness for the purposes of this study^2^.

We also opted to include two characteristics of openness which are not included as facets of openness to experience but that have been empirically demonstrated to be defining characteristics of openness: introspection (e.g., Goldberg, 1990, 1999; Connelly et al., 2014) and curiosity (e.g., Goldberg, 1990; Noftle et al., 2011). We therefore operationalized openness as a composite of traits that describe the tendency to explore ideas and emotions, to be introspective, curious, and aesthetically appreciative.

*Hypothesis 1:* Based upon the robust relationship between openness and creativity (e.g., Feist, 1998), we hypothesize that scores from the alternate uses tests administered after each daily exercise will be greater in the openness condition than in the control condition.
*Hypothesis 2*: Fleeson and Wilt (2010) have found that behaviors associated with openness are accompanied by elevated levels of state authenticity. Thus, we hypothesize that the experience of completing the daily experimental (openness) exercises will be accompanied by greater levels of authenticity than will the daily control exercises.
*Hypothesis 3:* Based on the relationship between openness and personal growth (e.g., Schmutte and Ryff, 1997), we hypothesize that individuals in the openness condition will report an increased inclination toward personal growth across pre-test and post-test time points as a result of the weeklong intervention.

These three hypotheses are informed by previous research. Given the exploratory nature of this experiment we have opted to conduct additional tests that relate directly or indirectly to isomorphism and openness. For example, although the relationship between positive affect and state extraversion does not seem to depend on dispositional levels of extraversion, the relationship between enacted openness and affect is not well-known, and potential interactions with dispositional openness are possible.

## Materials and Methods

### Participants

Two hundred and fifty-one participants enrolled in introductory psychology courses were recruited to participate in an online study. Two hundred and twenty-one cases were included in analyses. One hundred and six participants were randomly assigned to the openness condition (78% female; *M*_age_ = 21.82, *SD*_age_ = 4.58) and 115 participants were assigned to the control condition (73% female; *M*_age_ = 21.43, *SD*_age_ = 3.92). Thirty cases were excluded due to incomplete pre-test assessments (*n* = 8), failing to complete any daily logs (*n* = 13), and for concurrent participation in a separate but similar well-being intervention (*n* = 9). Participation was rewarded with course credit and the chance to win a $250 draw for completing daily logs, and/or a $250 draw for completing the optional follow-up survey. Our sample size was based on attrition rates and effect sizes from studies with a similar experimental design (publication forthcoming).

### Procedure

Participants registered for an online study entitled “Examining the relationship between well-being and cognition.” Following registration, participants were emailed a link to the pre-test survey which contained a several questionnaires and instructions for completing the remainder of the experiment. All participants were randomly assigned to condition.

For the 5 days following the pre-test survey, participants completed online daily logs. Daily logs 1–4 consisted of condition-specific writing tasks followed by a creativity assessment and a series of questionnaires assessing affect, authenticity, and effort experienced while completing the task. In lieu of a writing task, participants completed a trivia task in the fifth daily log.

A post-test assessment was administered the day following completion of the fifth daily log, and for those who opted-in, an additional follow-up survey was administered 2 weeks later. With the exception of a personality measure, the post-test and follow-up assessment contained an identical set of questionnaires as the pre-test assessment. A diagram of the experimental design is presented in **Figure 1**.

### Materials

#### IPIP-NEO-120

The IPIP-NEO-120 (Johnson, 2014) was created as public-domain alternative to Costa and McCrae (1992) NEO Personality Inventory (NEO PI-R) that assesses the five traits of the five factor model (openness, conscientiousness, extraversion, agreeableness, and neuroticism) as well as six lower-order facets for each trait. The IPIP-NEO-120 was administered at Time 1. Participants were presented with a list of 120 behavior descriptive statements/phrases such as “worry about things” and “believe in the importance of art” and were asked to indicate the degree to which each statement accurately describes them on a 1 (very inaccurate) to 5 (very accurate) Likert scale. Alpha coefficients for openness, conscientiousness, extraversion, agreeableness, and neuroticism were 0.80, 0.86, 0.86, 0.87, and 0.88, respectively.

#### Remote Association Test

The remote association test (RAT; Mednick, 1962) is a measure of creative thinking. Two separate versions of the RAT were administered during pre-test and post-test assessments. In each version participants were presented with a series of stimulus word triads (ex. AGE/ MILE/SAND) and prompted to provide a solution (ex. STONE) which, when paired with each stimulus word, would form compound words (ex. STONEAGE/ MILESTONE/SANDSTONE). Two 25-item versions of the RATs were compiled from a list of items developed by Bowden and Jung-Beeman (2003). Using the normative data for correct responses for each item provided by Bowden and Jung-Beeman (2003) we created two lists of 50 items each with closely match levels of difficulty. The RAT is scored by taking the sum of correct responses.

#### Meaning in Life Questionnaire

The meaning in life questionnaire (MILQ; Steger et al., 2006) is a 10-item self-report measure used to assess a subjective sense of meaning in life during pre-test, post-test, and follow-up assessments. The MILQ is comprised of two subscales: *search* and *presence*. The *presence* subscale (5-items) assesses the extent to which the respondent feels they have a sense of meaning in their life while the *search* subscale (5-items) assesses the extent to which the respondent is actively seeking to obtain a sense of meaning. On a 1 (absolutely false) to 7 (absolutely true) Likert scale participants rated items such as “I understand my life’s meaning” (*presence*) and “I am looking for something that makes my life feel meaningful” (*search*). Alpha coefficients were acceptable for both the *presence* (0.87) and *search* (0.89) subscales at Time 1.

#### Satisfaction With Life Scale

The satisfaction with life scale (SWLS; Diener et al., 1985) is a 5-item self-report measure designed to assess global life satisfaction and was administered at pre-test, post-test, and the follow-up assessments. On a 1–7 scale where 1 indicates strongly disagree and 7 indicates strongly agree, participants rated the extent to which they agree to the statements such as “In most ways my life is close to ideal” and “The conditions of my life are excellent.” The SWLS had an alpha coefficient of 0.88 at Time 1.

#### Alternative Uses Test

The alternative uses test (AUT; Christensen et al., 1960) assesses creative thinking by prompting respondents to list as many possible uses as they can for a common household object. The AUT was administered immediately following the condition-specific task in each daily logs. The five objects presented to participants in the five daily logs were *a brick*, *a newspaper*, *a paperclip, a pillow*, and *a shoebox.* Following scoring convention, each AUT response set was scored according to four components: *originality* (the relative uniqueness of each response)*, fluency* (total number of responses), *flexibility* (the number of conceptual categories), and *elaboration* (the amount of detail provided in each response). Originality scores were derived through assigned a score of 0–2 for each response; responses that comprised more than 5% of the total responses were assigned a score of 0, responses that made up less than 5% of the total responses were given a score of 1, and a score of 2 was assigned to each response which made up less than 1% of the total responses. Responses scored for elaboration were provided a value ranging from 0 to 2 which represented the degree of detail in each response. We scored each of the components while remaining blind to experimental condition.

#### Personal Growth

The psychological well-being scale (Ryff, 1989; Ryff and Keyes, 1995) is comprised of six subscales which assess the constructs of self-acceptance, environmental mastery, purpose in life, positive relations with others, autonomy, and personal growth. The 14-item personal growth subscale was administered at pre-test, post-test, and follow-up assessments. Using a 1 (strongly disagree) to 6 (strongly agree) Likert scale participants indicated the degree to which they agree with statements such as “For me, life has been a continuous process of learning, changing, and growth” and “I am the kind of person who likes to give new things a try.” The personal growth subscale is scored by calculating the mean response value across the 14-items; the alpha coefficient for the personal growth subscale was 0.88 at Time 1.

#### Authenticity and Effort Scale

The authenticity and effort scales were adapted from previous enacted trait research (Fleeson and Wilt, 2010; Gallagher et al., 2011) and recently validated (Smallenbroek et al., 2017). This measure assesses how authentic respondents feel in their behavior and the extent to which they feel that their behavior requires effort. Pre-test, post-test and follow-up assessments included a 15-item version of the measure (5 items for effort, 10 for authenticity) that assessed authentic and effortful behavior over the past week; a 7-item version was administered during each of the five daily logs (2 items for effort, 5 for authenticity) and assessed authentic and effortful behavior during the condition specific tasks. The authenticity and effort subscales were calculated by averaging response values contained within each subscale and demonstrated acceptable internal reliability at Time 1 (authenticity *a* = 0.86, effort *a* = 0.66).

#### The Positive and Negative Affect Schedule

The positive and negative affect schedule (PANAS-X; Watson and Clark, 1994) present a series of emotion descriptive adjectives and asks the respondent to indicate the extent to which their feelings are consistent with each item using a 1–5 likert scale where 1 indicates “very slightly or not at all” and 5 indicates “extremely.” A modified 45-item version of the 60-item PANAS-X was administered during pre-test, post-test, and follow-up assessments. The full PANAS-X is comprised of 60 items which are divided into four general categories: the *general dimensions*, the *basic negative emotions*, *basic positive emotions*, and *other affective states.* The 45-item version included all items of the PANAS-X except for those included in the basic negative emotions category, and instructed participants to respond in the context of how they have felt over the course of the past week. Alpha coefficients for the positive and negative affect subscales used in analyses were acceptable at Time 1 (positive affect *a* = 0.89; negative affect *a* = 0.84). A 27-item version (primarily comprised of the positive and negative subscales) was administered during each of the five daily logs. Participants were instructed to respond in the context of how they felt over the course of completing their condition specific activities.

#### Daily Logs Tasks (Experimental Condition)

Participants completed a series of tasks contained within the five online daily logs. The purpose of these tasks was to engage the individual characteristics of openness. When possible, tasks employed in previous research were adapted to fit the nature of the experiment. Tasks included a series of 15 min writing assignments that encouraged *introspection* and cognitive exploration of *aesthetics, ideas*, and *feelings* as well as a trivia task designed to elicit *curiosity*. During the pre-test assessment, participants were presented with the instructions in condensed form and asked to record their intended writing topics.

The first daily log addressed *aesthetic appreciation*. Instructions to reflect on aesthetics were adapted from “beauty logs” used in previous research (Diessner et al., 2006). Diessner et al. (2006) found that writing about natural, artistic and moral beauty in weekly “beauty logs” for 12 weeks lead to an increase in trait hope. Instructions, adapted from Diessner et al. (2006), prompted participants to spend 15 min writing about something beautiful that is (1) from nature; (2) man-made; and (3) in human-nature.

The second daily log addressed reflection on *feelings*. In order to facilitate reflecting on feelings, participants were prompted to spend 15 min writing down their deepest thoughts and emotions regarding two meaningful life events. Instructions were adapted from the emotional disclosure literature (Pennebaker and Francis, 1996; Lyubomirsky et al., 2006).

The third daily log addressed *introspection*. Reflecting upon personal characteristics via written exercises is a commonly employed task utilized in self-affirmation research and was thought to be a suitable introspective exercise. In these tasks participants are asked to rank order 11 values/characteristics (Cohen et al., 2000) in order of personal relevance and asked to explain (1) why their top ranked values/characteristics are important to them and (2) to specify a time then the value/characteristic was particularly important. Instructions were adapted from self-affirmation research (Cohen et al., 2000).

The fourth daily log addressed the exploration of *ideas*. A survey of relevant research did not yield an established method for inducing an exploration of ideas. Thus, instructions were crafted for this experiment. Participants were prompted to spend 15 min writing about one or two of the most interesting ideas/concepts that they have come across during their university experience. Specifically, participants were asked to briefly describe the idea/concept and elaborate on why they find it to be particularly interesting.

The purpose of the fifth log activity was to elicit a state of *curiosity*. Curiosity was elicited via the presentation of trivia items; a method derived from Kang et al. (2009). In order to observe the neurological effects of curiosity Kang et al. (2009) presented participants in an fMRI machine with a series of trivia items. For each presented item participants rated their level of curiosity and their confidence level that they knew the answer; forty trivia items and corresponding average participant ratings for each item were published in an **Supplementary Materials**. Participants in the present study were presented with 22 of these trivia items such as “what is the only country in the world where women dominate the government” and asked to rate on a 1 (not at all) to 7 (Likert scale) the degree to which they were curious to know the answer, and the extent to which they are confident they know the answer. The items presented to participants were those which had an average curiosity rating of four or above in the Kang et al. (2009) sample.

#### Daily Logs Tasks (Control Condition)

Across daily logs 1–4, participants in the control condition were asked to “record, in as much detail as possible, the happenings of your life in the past 24 h.” In the fifth daily log participants were presented with trivia items thought to elicit little or no curiosity in the average respondent. Two items, with low average curiosity ratings, were selected from the Kang et al. (2009) sample and 20 items were created for this experiment. Items such as “Who is the current Prime Minister of Canada?” and “H20 is the chemical compound for what substance” were crafted with the intent they would not elicit curiosity in the average respondent.

## Results

### Preliminary Analysis

In order to address the possibility that participants engaged with the activities to varying degrees depending on experimental condition, we compared the frequency of words written in the daily assignments between conditions. Word counts were computed in R Development (R Core Team, 2014) using the “stringi” package (Gagolewski and Tartanus, 2015). Overall participants wrote more words per writing exercise in the openness condition than in the control condition, however, effect sizes varied across time points (see **Table 1**). Due to this variation we included word counts as a predictor in all statistical models.

**Table 1:** Word count descriptive statistics for daily writing tasks 1–4.

	Openness			Control				
			
	n	*M*	*SD*	n	*M*	*SD*	*p*	*d*
Daily task 1	95	217.58	87.67	106	226.60	121.33	0.55	0.08
Daily task 2	99	270.27	115.75	102	176.24	115.24	<0.001	0.81
Daily task 3	93	158.47	84.95	99	168.10	117.59	0.52	0.09
Daily task 4	88	209.38	93.71	93	171.22	116.35	0.02	0.36
Overall	106	213.40	83.36	115	186.40	106.78	0.04	0.28

*Topics in the openness condition are aesthetics (Task1), emotions (Task 2), ideas (Task3), and introspection (Task4).*

As a manipulation check of the curiosity task we compared the average rating of how curious participants were to see the trivia answers between the openness and control condition. As expected those in the openness condition (*M* = 4.77, *SD* = 1.10) were more curious to know the answers to the trivia items than were those in the control condition (*M* = 3.20, *SD* = 1.39), *t*(184) = 8.45, *p* < 0.001, *d* = 1.25. Consistent with this finding, participants in the openness condition (*M* = 3.07, *SD* = 1.04) were less confident than participants in the control condition (*M* = 4.82, *SD* = 1.04) that they knew the answers to the trivia questions *t*(184) = 11.43, *p* < 0.001, *d* = 1.68. Thus, we can be relatively confident that the curiosity manipulation produced its intended effect.

### Analytic Strategy

We tested hypotheses with a multi-level modeling (MLM) approach using SAS 9.4. MLM allows for analysis of between group and within-person differences across time points in longitudinal designs. Variables were entered in a series of additive steps that were consistent across hypothesis tests. The first step was a random-intercept (or unconditional) model which partitions variance in the dependent variable into within person (level 1) and between person (level 2) variance. Variance estimates from the unconditional model were used to calculate the intraclass correlation (ICC). The ICC is computed using the level 1 and level 2 variance estimates from the unconditional model and represents the proportion of the total variance accounted for by level 2 variance (West et al., 2011). A second step was used to determine if Time should be considered a random effect in subsequent models, or in contrast, if a simpler model may provide a more reasonable fit (Singer, 1998). Next, several predictor variables were added into the model including the “condition” variable at level 2, the time-varying “word count” and time^∗^word count variables at level 1, and the time-invariant “trait openness” variable at level 2. With level 2 predictors substituted into the level 1 equation (i.e., full model expanded equation^3^), the following cross-level interaction terms were introduced into the model: time^∗^condition, word count^∗^condition, and time ^∗^trait openness. Word count was group centered and trait openness was grand mean centered.

Full Model: Random coefficients model with 4 predictors

Level 1: *y*_ti_ = β_0__i_ + β_1i_(time)_ti_ + β_2i_(word count)_ti_ + β_3i_ (time^∗^word count)_ti_ + *r*_ti_
Level 2: β_0i_ = *γ*_00_ + *γ*_01_(condition)_i_ + *γ*_02_(openness)_i_ + *γ*_03_(condition^∗^openness)_i_ + *u*_0i_
             β_1i_ = *γ*_10_ + *γ*_11_(condition)_i_ + *γ*_12_(openness)_i_ + *γ*_13_(condition^∗^openness)_i_ + *u*_1i_

             β_2i_ = *γ*_20_ + *γ*_21_(condition)
             β_3i_ = γ_30_

Full model expanded:

*y*_ti_ = *γ*_00_ + *γ*_01_(condition)_i_ + *γ*_02_(openness)_i_ + *γ*_03_(condition^∗^openness)_i_ + *γ*_10_(time)_ti_ + *γ*_11_(time^∗^condition)_ti_ + *γ*_12_(time^∗^openness)_ti_ + *γ*_13_(condition^∗^openness^∗^time)_ti_ + γ_20_(word count)_ti_ + *γ*_21_(word count^∗^condition)_ti_ + *γ*_30_(time^∗^word count)_ti_ + *u*_0i_ + u_1i_(time) + *r*_ti_

Because the presence of multiple non-significant interaction terms can unduly influence multilevel results, we employed a sequential testing strategy to remove non-significant interaction terms in order from largest to smallest *p*-values (see Aiken and West, 1991, p. 111–113). For brevity, this “final model” will be presented in this paper; full tables and further details on the analytical strategy can be found at https://osf.io/c5ejk/. Finally, all significant interactions involving a continuous variable were probed with simple slope analyses using scores of ±1SD relative to the mean (Aiken and West, 1991).

### Creativity

**FIGURE 2:**
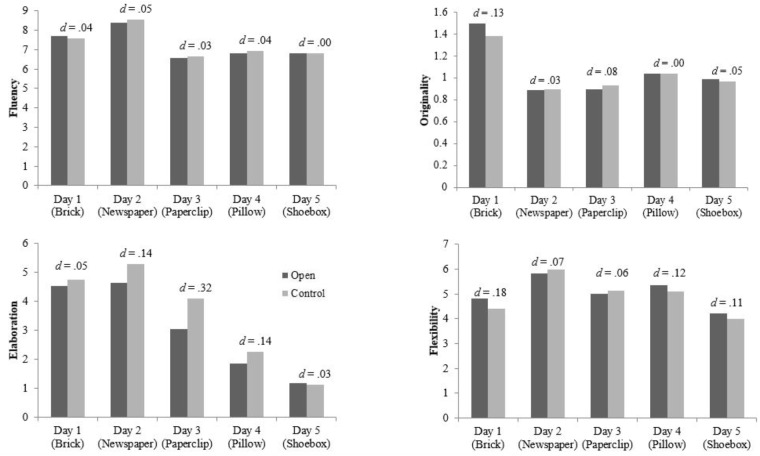
Raw mean scores for Alternate Uses Test components across daily assessments.

Hypothesis 1 proposes that scores on the alternate uses task would be greater in the openness condition than the control condition following the condition specific tasks for each of the four components of the alternate uses task: originality, flexibility (number of response categories), fluency (total number of response), and elaboration. Scores for each alternate uses task component are visualized in **Figure 2**; MLM results are presented in **Table 2**.

**Table 2:** MLM results for each Alternate Uses Test component.

Final model	Originality *B* (*SE*)	Fluency *B* (*SE*)	Elaboration *B* (*SE*)	Flexibility *B* (*SE*)
Intercept (γ_00_)	0.66 (0.08)	7.79 (0.65)^∗∗^	4.51 (0.72)^∗∗^	4.65 (0.42)^∗∗^
Time (γ_10_)	0.08 (0.02)	−0.36 (0.08)^∗∗^	−0.81 (0.10)^∗∗^	0.13 (0.06)^∗^
Condition (γ_01_)	0.04 (0.04)	0.26 (0.38)	0.78 (0.39)^∗^	0.09 (0.00)
Trait open (γ_02_)	0.11(0.04)^∗^	1.67 (0.42)^∗∗^	1.68 (0.42)^∗∗^	1.36 (0.27)^∗∗^
Word count (γ_20_)	0.0003 (0.0002)^∗^	0.0004 (0.002)	0.004 (0.001)^∗^	−0.002 (0.002)
Time^∗^Word count (γ_30_)	–	0.002 (0.0007)^∗^	–	0.002 (0.0005)^∗∗^
Intercept (τ_00_)	0.03 (0.01)^∗∗^	6.19 (0.75)^∗∗^	20.87 (3.16)^∗∗^	2.29 (0.31)^∗∗^
Time (τ_11_)	–	–	0.62 (0.23)^∗^	–
Cov (τ_10_)	–	–	−3.44 (0.78)^∗∗^	–
Residual (σ^2^)	0.10 (0.01)^∗∗^	4.38 (0.28)^∗∗^	6.18 (0.48) ^∗∗^	2.66 (0.17)^∗∗^
Intraclass correlation (ICC)	0.26	0.61	0.51	0.47

*^∗∗^p < 0.001; ^∗^p < 0.05. Dashes (“–“) denote instances where the variable was not included in the final model.*

MLM results revealed several notable findings. First, consistent with previous work on personality and creativity, trait openness predicted scores on the alternate uses task irrespective of condition. Second, and contrary to hypothesis 1, creativity scores were not greater in the openness condition than in the control condition^4^. For the elaboration component, a main effect of condition was detected suggesting that participants in the control condition provided more elaborate answers on average, β = 0.78, *SE* = 0.39, *t*(208) = 1.99, *p* = 0.05. Furthermore, elaboration scores were found to decrease significantly over time, β = −0.81, *SE* = 0.10, *t*(208) = −7.75, *p* < 0.001. Third, in each set of analyses the frequency of words written in the daily assessments predicted creativity scores (for originality and elaboration a main effect of word count was observed while a time by word count interaction was found for fluency, β = 0.002, *SE* = 0.0007, *t*(208) = 2.61, *p* = 0.01, and flexibility, β = 0.002, *SE* = 0.0005, *t*(208) = 3.49, *p* < 0.001). Simple slopes analyses revealed that individuals’ flexibility scores increased over time only for those who wrote more words than average in their writing tasks, β = 0.37, *SE* = 0.08, *t*(495) = 4.65, *p* < 0.001 (see **Supplementary Materials** for interaction plots involving word count). When probed, the fluency interactions suggest that fluency decreased significantly over time for those below the word count mean, β = −0.63, *SE* = 0.27, *t*(495) = −2.36, *p* = 0.02, however, this was not the case for those above the word count mean, β = −0.18, *SE* = 0.25, *t*(495) = −0.73, *p* = 0.47.

### Authenticity

Our second hypothesis predicted that the tasks in the open condition would elicit elevated levels of state authenticity when compared to the control group. As shown in **Table 3**, there was some small variation between conditions on authenticity across daily tasks (*d* = 0.05–0.17), however, the overall difference in authenticity was essentially zero (*d* = 0.01).

**Table 3:** Authenticity descriptive statistics (daily assessments).

	Openness			Control			
	n	*M*	*SD*	n	*M*	*SD*	*d*
Daily task 1	96	5.53	1.18	107	5.70	1.16	0.15
Daily task 2	99	5.76	1.35	104	5.54	1.31	0.17
Daily task 3	94	5.67	1.31	99	5.74	1.35	0.05
Daily task 4	89	5.79	1.26	93	5.66	1.47	0.09
Daily task 5	88	5.89	1.22	97	6.02	1.23	0.11
Overall	466	5.73	1.27	500	5.72	1.31	0.01

*Topics in the openness condition are aesthetics (task1), emotions (task 2), ideas (task3) and introspection (task 4); task 5 is the curiosity manipulation.*

**FIGURE 3:**
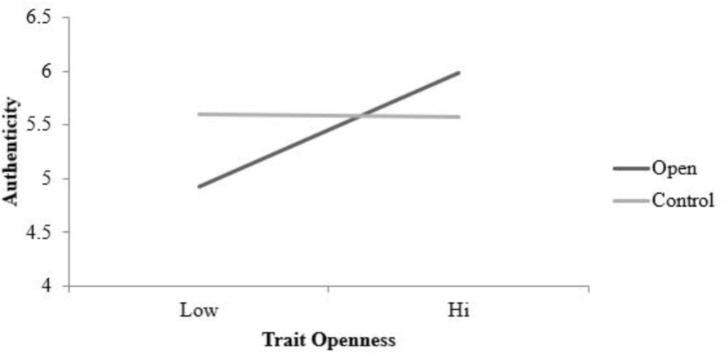
Simple slopes for condition (act open vs. control) by trait openness interaction predicting authenticity scores (daily assessments). The relationship between condition and authenticity depends on trait openness in the open condition, β = 1.18, *SE* = 0.25, *t*(216) = 4.64, *p* < 0.001 but not in the control condition, β = −0.02, *SE* = 0.26, *t*(216) = −0.09, *p* = 0.92 (final model). Condition lines are plotted for +1SD on trait openness. Authenticity scored on a 1–7 scale.

MLM results (see **Table 4**) revealed that the main effect of condition was not statistically significant, β = 0.02, *SE* = 0.03, *t*(216) = 1.40, *p* = 0.16, failing to support hypothesis 2. Interestingly, however, both the main effect of openness, β = 2.27, *SE* = 0.50, *t*(206) = 4.52, *p* < 0.001, and the trait openness by condition interaction were significant, β = 1.21, *SE* = 0.32, *t*(216) = 3.79, *p* < 0.001. Simple slopes analyses revealed that the relationship between condition and authenticity depended on trait levels of openness in the open condition, β = 1.18, *SE* = 0.25, *t*(216) = 4.64, *p* < 0.001 but not in the control condition, β = −0.02, *SE* = 0.26, *t*(216) = −0.09, *p* = 0.92. As shown in **Figure 3**, higher levels of trait openness were associated with elevated scores of daily authenticity in the open condition but not in the control condition.

**Table 4:** MLM models for authenticity across daily assessments.

Final model	B(SE)
Intercept (γ_00_)	5.49 (0.24)^∗∗^
Time (γ_10_)	0.05 (0.03)
Condition (γ_01_)	0.02 (0.14)
Word count (γ_20_)	0.002 (0.0005)^∗∗^
Trait open (γ_02_)	2.27 (0.50)^∗∗^
Trait open^∗^Condition(γ_03_)	−1.21 (00.32)^∗∗^
Intercept (τ_00_)	0.90 (0.18)^∗∗^
Time (τ_11_)	0.09 (.004)^∗∗^
Cov (τ_10_)	0.07 (0.05)
Residual (σ^2^)	0.50 (0.04)^∗∗^

*^∗∗^p < 0.001; ^∗^p < 0.05.*

### Personal Growth

**FIGURE 4:**
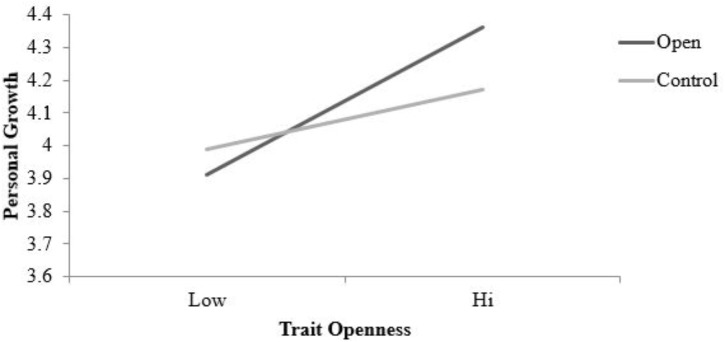
Simple slopes for condition (act open vs. control) by trait openness interaction predicting personal growth (pre-test, post-test. and follow-up assessments). Statistically significant effects were observed in both the control, β = 0.21, *SE* = 0.10, *t*(171) = 2.04, *p* = 0.04 and openness conditions, β= 0.50, *SE* = 0.10, *t*(171) = 4.88, *p* < 0.001 (final model). Condition lines are plotted for +1SD on trait openness. Personal growth scored on a 1–5 scale.

Descriptive statistics for personal growth scores, collected during the pre-test and post-test assessments, are presented in **Table 5**. In the final MLM model, a main effect of time was observed indicating that responses on the personal growth scale varied across time points, β = 0.05, *SE* = 0.02, *t*(206) = 2.60, *p* = 0.01. A significant main effect of word count suggests that individuals who wrote more during the daily writing tasks reported increasing higher scores on the personal growth scale pooling across the three time points, β = 0.0008, *SE* = 0.0003, *t*(206) = 2.38, *p* = 0.02. Consistent with previous findings (Schmutte and Ryff, 1997), trait openness was a significant predictor of personal growth when averaging across assessments in the final model, β = 0.21, *SE* =0.10, *t*(171) = 2.09, *p* = 0.04. A significant interaction between trait openness and condition also emerged, β = 0.33, *SE* = 0.14, *t*(171) = 2.44, *p* = 0.02. When probed, the trait openness by condition interaction revealed that the relationship between trait openness and personal growth scores was stronger for those in the experimental (openness) condition yet present in both the control, β = 0.21, *SE* = 0.10, *t*(171) = 2.04, *p* = 0.04 and openness conditions, β = 0.50, *SE* = 0.10, *t*(171) = 4.88, *p* < 0.001 (see **Figure 4**).

**Table 5:** Personal growth descriptive statistics (pre-test, post-test, and follow up assessments).

	Openness			Control			
	*n*	*M*	*SD*	*n*	*M*	*SD*	*D*
Pre-test	106	4.12	0.54	115	3.95	0.53	0.32
Post-test	86	4.11	0.57	93	4.14	0.57	0.05
Follow up	38	4.11	0.65	32	4.20	0.53	0.15

Several other variables were measured during the daily logs and the post-test assessments (see **Table 6**). However, the most relevant findings pertained to positive affect (descriptive statistics can be found in **Table 7** for daily logs).

**Table 6:** Summary of MLM results from the final model for daily logs assessing authenticity, effort, positive affect (PA), and negative affect (NA).

	Daily logs			
Final model	Authenticity *B* (*SE*)	Effort *B* (*SE*)	PA *B* (*SE*)	NA *B* (*SE*)
Intercept (γ_00_)	5.49 (0.24)^∗∗^	2.83 (0.26)^∗∗^	3.16 (0.17)^∗∗^	1.86 (0.20)^∗∗^
Time (γ_10_)	0.05 (0.03)	−0.12 (0.03)^∗∗^	−0.11 (0.02)^∗∗^	−0.12 (0.05)^∗^
Condition (γ_01_)	0.02 (0.14)	−0.12 (0.15)	−0.23 (0.10)^∗^	−0.11 (0.12)
Word count (γ_20_)	0.002 (0.0005)^∗∗^	0.003 (0.001)^∗^	0.001 (0.0003)^∗∗^	0.003 (0.0008)^∗∗^
Trait open (γ_02_)	2.27 (0.50)^∗∗^	−1.29 (0.54)^∗^	1.29 (0.36)^∗∗^	−0.07 (0.11)
Trait open^∗^ Condition(γ_03_)	−1.21 (0.32)^∗∗^	0.71 (0.34)^∗^	−0.80 (0.23)^∗∗^	–
Time^∗^Condition (γ_11_)	–	–	–	0.06 (0.03)^†^
Condition^∗^Word count (γ_21_)	–	−0.002 (0.001)^∗^	–	−0.001 (0.0006)^∗^
Intercept (τ_00_)	0.90 (0.18)^∗∗^	1.24 (0.24)^∗∗^	0.45 (0.09)^∗∗^	0.51 (0.03)^∗∗^
Time (τ_11_)	0.09 (0.04)^∗∗^	0.03 (0.02)^∗^	0.01 (0.01)	0.01 (0.01)^∗^
Cov (τ_10_)	−0.07 (0.05)	−0.07 (0.06)	−0.00 (.02)	−0.02 (0.02)
Residual (σ^2^)	0.50 (0.04)^∗∗^	0.65 (0.05)^∗∗^	0.26 (.02)^∗∗^	0.18 (0.01)^∗∗^
Inter class correlation (ICC)	0.61	0.62	0.59	0.71

*^∗^p < 0.05, ^∗∗^p < 0.001, ^†^p = 0.08. Dashes (“–“) denote instances where the variable was not included in the final model.*

**Table 7:** Positive affect descriptive statistics (daily assessments).

	Openness			Control			
	n	*M*	*SD*	n	*M*	*SD*	*d*
Daily task 1	96	2.86	0.80	107	2.64	0.87	0.26
Daily task 2	99	2.77	0.95	104	2.48	0.93	0.31
Daily task 3	94	2.70	0.83	99	2.38	0.95	0.36
Daily task 4	89	2.52	0.91	93	2.28	0.88	0.27
Daily task 5	88	2.60	0.86	97	2.57	0.82	0.04
Overall	466	2.69	0.88	500	2.48	0.90	0.24

*Topics in the openness condition are aesthetics (task 1), emotions (task 2), ideas (task 3) and introspection (task 4); task 5 is the curiosity manipulation.*

**FIGURE 5:**
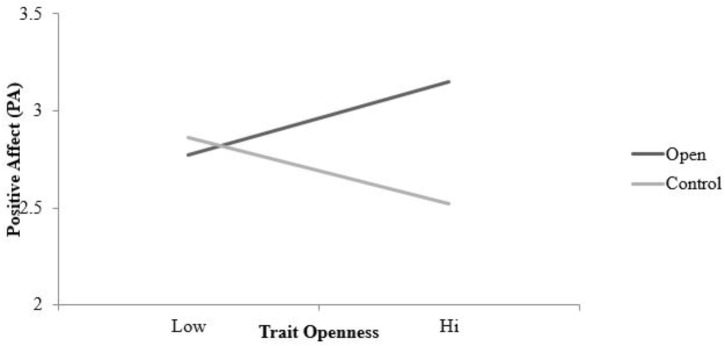
Simple slopes for condition (act open vs. control) by trait openness interaction predicting positive affect (daily assessments). Simple slopes were statistically significant in both conditions, but with opposite signs: in the open condition, β = 0.43, *SE* = 0.18, *t*(216) = 2.34, *p* = 0.02, and in the control condition, β = −0.37, *SE* = 0.18, *t*(216) = −2.01, *p* = 0.05 (final model). Condition lines are plotted for +1SD on trait openness. Positive affect scored on a 1–5 scale.

Interestingly, positive affect was found to vary across daily logs^5^ β = −0.11, *SE* = 0.02, *t*(548) = −5.52, *p* < 0.001, such that individuals reported less positive affect as the study progressed. A main effect of condition was also found, β = −0.23, *SE* = 0.10, *t*(216) = −2.12, *p* = 0.03, indicating that the participants reported more positive affect immediately following the open condition activities then the control condition activities. A main effect of trait openness was observed suggesting that higher levels of trait openness were associated with more positive affect averaging across assessments, β = 1.29, *SE* = 0.36, *t*(216) = 3.58, *p* < 0.001. A statistically significant condition by trait openness interaction was also found, β = 0.79, *SE* = 0.23, *t*(216) = 3.50, *p* < 0.001. The simple slope for the open condition, β = 0.43, *SE* = 0.18, *t*(216) = 2.34, *p* = 0.02, and the control condition, β = −0.37, *SE* = 0.18, *t*(216) = −2.01, *p* = 0.05, were both statistically significant, but opposite in sign. As illustrated in **Figure 5**, high levels of dispositional openness were associated with greater positive affect in the openness condition and lower positive affect in the control condition.

## Discussion

This experiment investigated the outcomes of behavior associated with open states. Guided by previous enacted trait research (e.g., Zelenski et al., 2012, 2013) and the trait-state isomorphism hypothesis (Fleeson, 2001), we proposed two hypotheses regarding the outcomes of open states. Specifically, we hypothesized that engaging in open behavior, when compared to a control group, would be associated with creative thinking ability, elevated levels of authenticity. We failed to find support for these hypotheses. Furthermore, results did not demonstrate a relationship between open behavior and changes in personal growth over time. This lack of support for the trait-state isomorphism of openness contrasts with cases where traits and states are characterized in part by similar outcomes (e.g., extraversion and positive affect; neuroticism and negative affect). Additionally, and also contrasting with previous enacted trait research, we observed several instances where dispositional openness was associated with more positive outcomes in the openness condition than the control condition (i.e., personal growth, positive affect, authenticity).

### Creativity

Our results supported typical findings in the literature suggesting that trait openness is a significant predictor of creative thinking ability (e.g., Feist, 1998). However, in contrast with the isomorphism hypotheses, momentary open behaviors did not influence creativity significantly. Unexpectedly, main effects of time were observed across each of the four components of the alternate uses task. The contrasting directions of trends over time provide insight into potential shortcomings of the experimental design. For example, the fluency and elaboration scales of the alternate uses task reflect the amount and level of detail, respectively, both fluency and elaboration scores decreased over the five time points. In that these components are more quantitative rather than qualitative (cf. the originality and flexibility components), the decrease in elaboration and fluency over time may best be accounted for by motivational effects. That is, it is likely that participants gave more effort to the first assessments than the fifth. Changes to experimental design elements in future studies may counteract this motivational effect.

An unexpected finding was the role of word count in predicting scores on each of the four alternate use task components (across conditions). In that word counts in writing exercises can be interpreted as a proxy for effort (e.g., Layous and Lyubomirsky, 2012), the set of findings observed in this experiment may simply suggest that individuals who put forth a greater effort in the daily tasks also put more effort into the creative thinking assessment and subsequently scored higher on the alternate uses task. Alternatively, it is conceivable that the writing process itself is conducive to creative thinking. This rationale is often used to promote the use of “free-writing” or “wet-ink” exercises. Although a literature review did not reveal empirical tests of the link between free-writing and creative thinking ability, it is believed that the process of continuous writing is associated with creative thinking ability (e.g., Bonk and Smith, 1998).

In summary, although evidence supported the relationship between trait openness and creative thinking ability, this experiment failed to find support for the notion that manipulated behaviors and cognitions associated with trait openness facilitate creative thinking ability.

### Authenticity

We predicted that states of openness would be associated with ratings of authenticity, i.e., the evaluation that one’s behavior is in line with their sense of “true self.” This hypothesis was informed by experience sampling research which has demonstrated the link between open states and authenticity (Fleeson and Wilt, 2010). Given that open states have been found to covary with assessments of authentic behavior, one would expect to find that open states in the present study would also show this relationship with authenticity.

Authenticity scores did not differ significantly between conditions, however, this lack of effect could possibly be due to ill-suited control condition activities. Although the control group was a reasonable comparison for the majority of the variables of interest, it may be that writing about 1 day also feels authentic. Alternatively, feelings of authenticity may be more likely to occur when the open behaviors are selected by the participants and enacted in naturalistic settings (e.g., Fleeson and Wilt, 2010) than when assigned as part of a formal experiment.

Interestingly, trait openness was associated with feeling authentic while completing the daily tasks in general, however, the trait openness by condition interaction suggests that this effect was much stronger in the open condition.

### Influence of Trait Openness

In three notable instances, trait openness by condition interactions were observed; these interactions predicted personal growth (post-test assessments), authenticity (daily assessments), and positive affect (daily assessments). In each case, open individuals in the open condition reported more positive/desirable outcomes than their less open peers. This contrasts with previous enacted trait research wherein dispositional extraversion and neuroticism do not impact the outcomes associated with extraverted and neurotic behavior (i.e., positive and negative affect; Fleeson and Wilt, 2010). There are at least two plausible explanations that account for this pattern of results.

First, trait openness is often considered an “enhancing” or “amplifying” factor. For example, individuals high on trait openness typically report the greatest benefit in positive psychology interventions (Senf and Liau, 2012). Openness has also been found to amplify the association between extraversion and well-being, although openness, similarly, amplifies the relationship between neuroticism and distress (Bardi and Ryff, 2007). Thus, it seems plausible that trait openness likewise played an amplifying role for participants in the open condition. That is, the open condition consisted of a number of cognitively engaging activities and participants high on trait openness were more sensitive to the potential positive benefits of these activities.

Second, it seems equally plausible that acting in accordance with one’s trait would lead to desirable outcomes. The idea that trait-consistent behavior produces more enjoyment is consistent with the behavioral concordance model (Moskowitz and Cote’, 1995). Despite the intuitive appeal of behavioral concordance model, findings from the enacted trait literature suggest that behaving in a manner which is out of character can in fact be enjoyable. Specifically, findings from experience sampling studies and laboratory experiments demonstrate that acting in an extraverted manner is enjoyable even for dispositional introverts. However, in contrast with enacted trait research on extraversion, the results of this experiment arguably support the notion that engaging in trait congruent behavior is enjoyable for open individuals but not their less open counterparts. Potential support for this proposition is particularly evident with the positive affect results (see **Figure 5**). Higher levels of trait openness were associated with higher levels of positive affect experienced during the condition-specific activities, while lower levels of trait openness predicted lower levels of positive affect. Thus, it is possible that acting in accordance with one’s disposition, when considering trait openness, is enjoyable and beneficial, while acting counter to one’s trait is markedly less enjoyable and beneficial.

The interactions between levels of trait openness and experimental condition in this study can arguably be explained by two competing interpretations. That is, it is possible that open individuals respond more strongly to positive activities in general, and it is also possible that engaging in trait congruent behavior leads to beneficial/enjoyable outcomes. Future research is needed to further assess these competing explanations in relation to trait openness. Additionally, subsequent attempts to manipulate openness could consider whether other stable individual differences (e.g., intelligence) also influence outcomes associated with enacted openness.

### State-Trait Isomorphism

This experiment tested a set of hypotheses predicted by the notion of trait-state isomorphism. Experiencing sampling studies and experimental manipulation have demonstrated that both extraversion and neuroticism are at least partially isomorphic. That is, extraversion is associated with positive affect at both the trait and state level, and neuroticism is likewise associated with negative affect. Interestingly, with the exception of positive affect, no evidence of isomorphism was observed with openness and creative thinking ability or personal growth.

One salient pattern of results across isomorphism studies is that affective outcomes appear to be subject to isomorphism while there is no evidence thus far that more cognitive outcomes such as creativity or personal growth are isomorphic. That is, the relationship between openness and positive affect observed at the trait level was also observed at the state level in the present experiment. This isomorphism with an affective outcome is consistent with the established findings concerning state extraversion and neuroticism. However, the relationship between trait openness and creative thinking ability was not observed at the state level. Future research is needed to understand whether or not the affect-cognition distinction ultimately explains our pattern of results well.

### Limitations and Future Research

This research project represents the first (known) empirical attempt to manipulate states of openness as a means of further testing the trait-state isomorphism hypothesis. However, this initial exploratory study is subject to a number of limitations.

First and foremost, the lack of precision surrounding the exact composition of behaviors which comprise openness complicates any attempt to manipulate open states. Although each of the components of openness in this experiment (aesthetics, ideas, emotions, introspection, and curiosity) are all considered to be facets of openness in some taxonomies, this exact composition was not derived from a single taxonomy but rather was assembled from various taxonomies in order to address conceptual issues with the most popular personality inventory, the NEO-IP-R^6^ (Costa and McCrae, 1992). Even among well-validated personality taxonomies, there is little consensus on a precise assortment of open facets. Although clearly defining specific facets in enacted trait research allows for sufficient testing of the isomorphism hypothesis, until this issue is resolved enacted trait research with openness will continue to be subject to this limitation.

With this limitation in mind, this experiment consisted of five activities designed to elicit states of aesthetic appreciation, emotional exploration, abstract conceptual exploration, introspection, and curiosity. With the exception of the exploration of ideas activity, all activities were successfully employed in the literature previous to their use in this experiment. Upon reflection on these activities, we would recommend two alterations for future research. Frist, the introspection activity was adapted from the self-affirmation literature (Cohen et al., 2000). Given its original source, the task encourages self-affirming introspection and leaves little room for introspection on less positive features of oneself. This bias limits scope in that introspection can be both self-affirming and non-affirming. Second, state openness measures could be administered following each activity in order to verify the manipulations produced the intended effect. That is, without explicit manipulation checks we cannot empirically verify that open states were produced in this sample (though curiosity was confirmed). For most activities, previous work suggests their effectiveness in this regard; nonetheless, future research might seek to verify these states if only to increase the confidence in other null results (e.g., in creativity). However, the potential costs and benefits of including manipulation checks should be carefully considered (Sigall and Mills, 1998; Fayant et al., 2017). In the present study, we opted not to conduct manipulation checks for the daily tasks. Due to the fact that participants engaged in behaviors associated with trait openness they were, by definition, in open states. Although we cannot be certain that participants were in “highly open” states, it is likely the activities promoted open states, particularly when compared to the control condition.

Future research may also benefit from conducting enacted openness studies in a laboratory setting. Results from the creativity assessments strongly suggest that participants’ motivation to fully engage in the study decreased as time progressed. Because research has demonstrated that the perception of being observed significantly enhances cooperative behavior (e.g., Bateson et al., 2006; Ernest-Jones et al., 2011), one potential method of counteracting motivational effects may be to conduct in-lab experiments. The lab environment might also provide a statistically more powerful test of isomorphism by reducing variability in how and where participants enact behaviors.

## Conclusion

This experiment sought to test the degree to which the outcomes of trait and state openness are isomorphic. Contrary to the isomorphism hypothesis, open behaviors did not influence creative thinking ability. Thus, the promise of enacted openness as a method of facilitating creative thinking was not supported. Additionally, open states were not found to be associated with higher levels of authenticity relative to a control group (although dispositional openness predicted authenticity), and results did not support the link between open behavior and personal growth. However, several unexpected trait by condition interactions were observed indicating that the dispositionally open benefited more from open states, when compared to the control condition. This pattern diverges from previous findings in the enacted trait literature (e.g., where dispositional extraversion does not influence the relationship between state extraversion and positive affect). Therefore, our findings suggest that disposition may, in some instances, influence the outcomes associated with trait-related behavior.

This study represents the first empirical attempt to manipulate states of openness and presents preliminary evidence for the relative lack of trait-state isomorphism for openness. Although we recognize some method limitations, it is still striking that little evidence for isomorphism emerged. That is, across five manipulations of open states no concrete, or even modestly suggestive, evidence was observed to indicate that state openness was associated with creative thinking ability. Positive affect was observed to be partially isomorphic, however, this could conceivably be due to either trait-state isomorphism or differences in the inherent pleasantness of condition specific tasks. When these findings are compared to the existing literature it becomes apparent that only affective outcomes have been found to be isomorphic, while cognitive characteristics such as creativity do not appear to be isomorphic. Thus, results from this experiment tentatively suggest trait-state isomorphism may not extend to cognitive outcomes. More research is needed to confirm this, and then to understand the underlying mechanisms and processes which renders some traits (e.g., extraversion and neuroticism) to be partially isomorphic and others seemingly less isomorphic (e.g., openness).

## Ethics Statement

This study was carried out in accordance with the recommendations of the Carleton University Ethics Board with written informed consent from all subjects. All subjects gave written informed consent in accordance with the Declaration of Helsinki. The protocol was approved by the Carleton University Ethics Committee.

## Author Contributions

ZvA and JZ conceived of the ideas presented in the manuscript as part of ZvA’s masters thesis. ZvA drafted the manuscript with assistance from JZ.

## Conflict of Interest Statement

The authors declare that the research was conducted in the absence of any commercial or financial relationships that could be construed as a potential conflict of interest.

## Footnotes

1 We refer to “openness to experience” when discussing the conceptualization developed by Costa and McCrae while using the term “openness” to refer to the fifth factor more broadly (for ease of communication).

2 A more complete argument for our selection of facets can be found in **Supplementary Materials**.

3 Notations [with explanations] for the model are as follows: *y* [score on dependent variable]; _ti_ [variable takes on different values for each observation _t_ for each person _i_]; β_0i_ [intercept of dependent variable for individual _i_]; β_1i,_ – β_3i_ [slope for person _i_ between level 1predictor and dependent variable]; *r* [residual]; *γ*_00_ [overall intercept or grand mean]; *γ*_01−_ *γ*_03_ [slope between dependent variable and level 2 predictor]; *u*_0i_ [random error of the intercept]; *γ*_01−_ *γ*_13_ [slope between dependent variable and level 1 predictor]; *u*_1i_ [random error of slope].

4 In order to test whether the intervention produced any long term effects on creative thinking ability a 2 (condition) × 2 (time) mixed-factors analysis of variance (ANOVA) was conducted with remote association task scores as the dependent variable. No main effect was observed for condition, *F*(1, 134) = 0.42, *p* = 0.52, partial η^2^ = 0.00, and the condition by time interaction was not significant, *F*(1, 134) = 0.005, *p* = 0.92, partial η^2^ = 0.00.

5 Results from post-test assessments can be found in **Supplementary Materials**.

6 See **Supplementary Materials** for the rationale employed in the facet selection.
