# Supplementary material for: Testing Trait-State Isomorphism in a New Domain: An Exploratory Manipulation of Openness to Experience
Source: Front Psychol. 2018 Oct 15;9:1964. doi: 10.3389/fpsyg.2018.01964 (PMC6232896; doi:10.3389/fpsyg.2018.01964)
Supplement: Supplementary file 1 [file Table_1.docx]

**Rationale for inclusion/exclusion of openness facets**

Given the lack of consensus regarding the defining characteristics of openness I arrived at a selection of characteristics rationally. As a starting point I began with Costa and McCrea’s (1992) conceptualization of openness to experience as it represents the ‘mainstream’ view of openness. The six facets of openness to experience include: ideas, aesthetics, values, feelings, actions, and fantasy.
 Research clearly demonstrates the aesthetics and ideas facets to be the best markers of openness (e.g. Johnson, 1994). Additionally, evidence supports the notion that open individuals are more sensitive to and aware of their internal emotional states (e.g. Connelly et al., 2015, Sobocko & Zelenski, 2015). Thus, the preference for ideas, the appreciation of aesthetics, and emotional awareness were retained as defining characteristic of openness for the purposes of this study.
 The remaining three facets of actions, values, and fantasy however, were not retained. DeYoung, Peterson and Higgins (2005) observed that of all of the NEO PI-R facet of openness, the actions facet was the least correlated with trait openness to experience (*r* = .40) and the most related to trait extraversion (*r* = .44). Therefore, insofar as openness to experience represents abstract/cognitive exploration and extraversion represents concrete/behavioural exploration the ‘actions’ facet, with its emphasis on behavioural flexibility, may not be an ideal marker of openness (DeYoung et al., 2005, DeYoung et al., 2014).

Trait openness is negatively correlated with political conservatism, dogmatism, and right-wing authoritarianism (McCrae & Costa, 1997) and the values facet of openness to experience represents openness to liberal values rather than values in general. For example the open source version of the NEO-IP-R, the IPIP, re-labeled the values facet ‘liberalism’ and included items such as “tend to vote for liberal political candidates”. However, recent research suggests that political orientation is best associated with a combination of openness, agreeableness (especially the politeness rather than compassionate aspect; Hirsh, DeYoung, Xu, & Peterson, 2010) and the orderliness components of conscientiousness (Carney, Jost, Gosling, & Potter, 2008). Thus, among other reasons, manipulating cognitions associated with political liberalism as a manipulation of openness is problematic given its relationship with three of the big five traits.
 The tendency to engage in fantasy was omitted as a characteristic of openness for pragmatic reasons. Wilson et al (2014) conducted several studies that involved ‘prompted fantasy’ and found that participants often had difficulty following the instructions when in the lab or at home and over 30% of participants self-reported cheating on the task by engaging with external stimuli such as cell phones and laptops. Thus, given the variety of characteristics empirically demonstrated to partially define the fifth factor, and the likely prospect that a prompted fantasy manipulation would be problematic, I decided to exclude fantasy as a characteristic of openness for the purposes of the manipulation.
 Finally, I opted to include two characteristics of openness which are not included as facets of openness to experience but that have been empirically demonstrated to be defining characteristics of openness: introspection (e.g. Connelly et al., 2015; Goldberg, 1990; Goldberg, 1999 ) and curiosity (e.g. Goldberg, 1990; Noftle, Schnitker, & Robins, 2011). Thus, I operationalized openness as a composite of traits that describe the tendency to explore ideas and emotions, to be introspective, curious, and aesthetically appreciative.

## Statistical Approach

With one exception, I elected to test all hypotheses though a Multi-Level Modelling (MLM) approach using SAS 9.4. MLM allows for analysis of between group and within-person differences across time points in longitudinal designs. Given the nature of my data MLM confers a number of advantages over other statistical options such as t-tests, regression, or Analysis of Variance tests (ANOVA). First, the number of t-tests required to address my entire set of hypothesis would require steep p-value corrections to adjust for family-wise error; however, MLM arguably addresses this issue (Gelman, Hill, & Yajima, 2012). Second, I am interested in trait by condition interactions. These categorical by condition interactions are normally testable with regression methods; however, because I am interested in these interactions across time points MLM is required. Third, the unequal spacing between the pretest, posttest, and follow-up assessments violated an assumption of the general linear model. In contrast, MLM is not subject to this restriction (Singer, 1998).

In constructing models I used a series of additive steps that were consistent across hypothesis tests. Model A represents a random-intercept (or unconditional) model which will partition variance in the dependent variable into within person (Level 1) and between person (Level 2) variance.

*Model A: Random intercept (unconditional) model*

Level 1: *y_ti_* = β_0_*_i_* + *r_ti_*

Level 2: β_0_*_i_ = γ_00_ + µ_0i_*

Variance estimates from the unconditional model can also be used to calculate the intraclass correlation (ICC). The ICC is computed using the Level 1 and Level 2 variance estimates from the unconditional model and represents the proportion of the total variance accounted for by Level 2 variance (West, Ryu, Kwok, & Cham, 2011).

*Intraclass Correlation*

ICC = $\frac{\tau00}{\tau00+\sigma2}$

Model B is identical to Model A however the variable ‘time’ was allowed to vary randomly. The results from this model determine whether Time should be considered a random effect in subsequent models, or in contrast, if a simpler model may provide a more reasonable fit (Singer, 1998).

Model C is a random coefficients model which adds the level 2 predictor of ‘condition’ and a condition by time interaction to test whether potential variation over time occurs at different rates depending upon condition.

*Model C: Random coefficients model with 2 predictors (Time and Condition)*

Level 1: *y_ti_* = β_0_*_i_* + β_1i_(Time)_ti_ + *r_ti_*

Level 2: β_0_*_i_ = γ_00_ + γ_01_(*Condition*)_i_* + *µ_0i_*

β_1_*_i_ = γ_00_ + γ_11_(*Condition*)_i_* + *µ_0i_*

*Full model expanded:*

*y_ti_* = *γ_00_* + *γ*_10_(Time)_ti +_ *γ_01_(*Condition*)_i_ + γ_11_*(Time_*_Condition) _ti_

+ *µ_0i_ + µ_1i_*(Time) + *r_ti_*

In Model D I added the time-varying predictor variable of ‘word count’ (group centered) and time*word count interaction as a level 1 predictors and the time-invariant variable ‘trait openness’ (group centered) as a level 2 predictor.

*Full Model: Random coefficients model with 4 predictors*

Level 1: *y_ti_* = β_0_*_i_* + β_1i_(time)_ti_ + β_2i_(word count) + β_3i_ (time*word count) + *r_ti_*

Level 2: β_0_*_i_ = γ_00_ + γ_01_(*condition*)_i_* + *γ_02_(*openness*)_i_* + *γ_03_(*condition*openness) *_i_*
 + *u_0i_*

β_1_*_i_ = γ_01_ + γ_11_(*condition*)_i_* + *γ_12_(*openness*)_i_* + *γ_13_(*condition***openness)*_i_* + *u_1i_*

β_2_*_i_* = *γ_20_* + *γ_21_*(condition)

β_3_*_i_* = *γ_30_*

*Full model expanded:*

*y_ti_* = *γ_00_* + *γ_01_(*condition*)_i_* + *γ_02_(*openness*)_i_*+ *γ_03_(*condition***openness)*_i_*

*+ γ*_10_(time)_ti_ *+ γ_11_*(time*condition)_ti_ + γ_12_(time*openness) _ti_

+ *γ*_10_(condition*openness*time) _ti_

+ *γ*_20_(word count)_ti_+ *γ_12_*(word count*condition) _ti_

+ γ_30_(time*word count) _ti_

+*u_0i_ + u_1i_*(time) + *r_ti_*

Finally, in all cases a Model E is presented. Because the presence of multiple non-significant interaction terms can unduly influence multilevel results, I employed a sequential testing strategy to remove non-significant interaction terms in order from largest to smallest p-values (see Aiken & West, 1999, pp. 111-113).

**Supplementary Tables and Figures**

|  | Openness | | Control | |  |
| --- | --- | --- | --- | --- | --- |
|  | *M* | *SD* | *M* | *SD* | *d* |
| Day 1 (Brick) | 1.50 | .89 | 1.38 | .89 | .13 |
| Day 2 (Newspaper) | 0.89 | .35 | 0.90 | .37 | .03 |
| Day 3 (Paperclip) | 0.90 | .37 | 0.93 | .35 | .08 |
| Day 4 (Pillow) | 1.04 | .40 | 1.04 | .39 | .00 |
| Day 5 (Shoebox) | 0.99 | .41 | 0.97 | .40 | .05 |

Table 2. Alternate uses task ‘originality’ descriptive statistics (daily assessments). Topics in the openness condition are aesthetics (task 1), emotions (task 2), ideas (task 3) and introspection (task 4); Task 5 is a curiosity manipulation.

Figure 4. Mean alternate uses task ‘originality’ scores over time (daily assessments). Topics in the openness condition are aesthetics (task 1), emotions (task 2), ideas (task 3) and introspection (task 4).

| Model |  | Parameter Estimates | Standard Error | *t* | *z* | *p* |
| --- | --- | --- | --- | --- | --- | --- |
|  |  |  |  |  |  |  |
| A | Intercept (γ_00_) | 0.81 | .04 | 18.91 |  | <.001 |
|  | Time (γ_10_) | 0.04 | .01 | 3.62 |  | <.001 |
|  | Intercept (τ_00_) | 0.04 | .01 |  | 5.15 | <.001 |
|  | Residual (σ^2^) | 0.11 | .00 |  | 15.76 | <.001 |
|  |  |  |  |  |  |  |
| B | Intercept (γ_00_) | 0.81 | .04 | 19.33 |  | <.001 |
|  | Time (γ_10_) | 0.04 | .01 | 3.48 |  | <.001 |
|  | Intercept (τ_00_) | 0.03 | .04 |  | 0.73 | .234 |
|  | Time (τ_11_) | 0.00 | .00 |  | 0.61 | .272 |
|  | Cov (τ_10_) | -0.00 | .01 |  | -0.20 | .839 |
|  | Residual (σ^2^) | 0.11 | .01 |  | 12.72 | <.001 |
|  |  |  |  |  |  |  |
| C | Intercept (γ_00_) | 0.73 | .14 | 5.32 |  | <.001 |
|  | Time (γ_10_) | 0.06 | .04 | 1.70 |  | .090 |
|  | Condition (γ_01_) | 0.05 | .09 | 0.60 |  | .546 |
|  | Time*Condition (γ_11_) | -0.01 | .22 | -0.60 |  | .548 |
|  | Intercept (τ_00_) | 0.04 | .01 |  | 5.16 | <.001 |
|  | Residual (σ^2^) | 0.11 | .01 |  | 15.74 | <.001 |
|  |  |  |  |  |  |  |
| D | Intercept (γ_00_) | 0.62 | .19 | 3.27 |  | .001 |
|  | Time (γ_10_) | 0.09 | .06 | 1.53 |  | .128 |
|  | Word Count (γ_20_) | -0.00 | .00 | -0.25 |  | .799 |
|  | Condition (γ_01_) | 0.07 | .12 | 0.57 |  | .571 |
|  | Trait Open (γ_02_) | 0.08 | .17 | 0.45 |  | .650 |
|  | Trait Open*Condition (γ_03_) | -0.04 | .08 | -0.45 |  | .655 |
|  | Trait Open*Time (γ_12_) | 0.03 | .04 | 0.73 |  | .466 |
|  | Time*Condition (γ_11_) | -0.01 | .04 | -0.29 |  | .771 |
|  | Time*Word Count (γ_30_) | 0.00 | .00 | 1.20 |  | .229 |
|  | Condition*Word Count(γ_21_) | -0.00 | .00 | -0.06 |  | .953 |
|  | Intercept (τ_00_) | 0.03 | .01 |  | 4.04 | <.001 |
|  | Residual (σ^2^) | 0.10 | .01 |  | 12.64 | <.001 |
|  |  |  |  |  |  |  |
| E | Intercept (γ_00_) | 0.66 | .08 | 8.10 |  | <.001 |
|  | Time (γ_10_) | 0.08 | .02 | 4.39 |  | <.001 |
|  | Word Count (γ_20_) | 0.00 | .00 | 2.29 |  | .023 |
|  | Condition (γ_01_) | 0.04 | .04 | 0.96 |  | .339 |
|  | Trait Open (γ_02_) | 0.11 | .04 | 2.59 |  | .010 |
|  | Intercept (τ_00_) | 0.03 | .01 |  | 4.04 | <.001 |
|  | Residual (σ^2^) | 0.10 | .01 |  | 12.72 | <.001 |
|  |  |  |  |  |  |  |

Table 3. MLM models for alternate uses task ‘originality’ (daily assessments). Topics in the openness condition are aesthetics (task 1), emotions (task 2), ideas (task 3) and introspection (task4).

|  | Openness | | Control | |  |
| --- | --- | --- | --- | --- | --- |
|  | *M* | *SD* | *M* | *SD* | *d* |
| Day 1 (Brick) | 4.83 | 2.19 | 4.42 | 2.27 | .18 |
| Day 2 (Newspaper) | 5.82 | 2.37 | 5.99 | 2.69 | .07 |
| Day 3 (Paperclip) | 5.02 | 2.26 | 5.14 | 2.10 | .06 |
| Day 4 (Pillow) | 5.36 | 2.30 | 5.09 | 2.26 | .12 |
| Day 5 (Shoebox) | 4.23 | 1.96 | 4.01 | 2.04 | .11 |

Table 4. Alternate uses task means and SDs for flexibility. Topics in the openness condition are aesthetics (task 1), emotions (task 2), ideas (task 3) and introspection (task 4); task 5 is the curiosity manipulation.

Figure 5. Mean alternate uses task ‘flexibility’ scores over time (daily assessments). Topics in the openness condition are aesthetics (task 1), emotions (task 2), ideas (task 3) and introspection (task 4).

Figure 6. Simple slopes for time by word count interaction predicting alternate uses tasks ‘flexibility’ scores (daily assessments).

| Model |  | Parameter Estimates | Standard Error | *t* | *z* | *p* |
| --- | --- | --- | --- | --- | --- | --- |
|  |  |  |  |  |  |  |
| A | Intercept (γ_00_) | 5.40 | .17 | 31.27 |  | <.001 |
|  | Time (γ_10_) | -0.17 | .04 | -4.25 |  | <.001 |
|  | Intercept (τ_00_) | 2.54 | .32 |  | 7.91 | <.001 |
|  | Residual (σ^2^) | 2.86 |  |  |  |  |
|  |  |  |  |  |  |  |
| B | Intercept (γ_00_) | 5.39 | .18 | 30.03 |  | <.001 |
|  | Time (γ_10_) | -0.17 | .04 | -4.18 |  | <.001 |
|  | Intercept (τ_00_) | 3.04 | .57 |  | 5.33 | <.001 |
|  | Time (τ_11_) | - | - |  | - | - |
|  | Cov (τ_10_) | -0.08 | .07 |  | -1.14 | .253 |
|  | Residual (σ^2^) | 2.86 | .16 |  | 18.38 | <.001 |
|  |  |  |  |  |  |  |
| C | Intercept (γ_00_) | 5.49 | .56 | 9.82 |  | <.001 |
|  | Time (γ_10_) | -0.15 | .13 | -1.16 |  | .248 |
|  | Condition (γ_01_) | -0.06 | .35 | -0.18 |  | .860 |
|  | Time*Condition (γ_11_) | -0.01 | .08 | -0.16 |  | .870 |
|  | Intercept (τ_00_) | 2.55 | .32 |  | 7.90 | <.001 |
|  | Residual (σ^2^) | 2.87 | .16 |  | 18.36 | <.001 |
|  |  |  |  |  |  |  |
| D | Intercept (γ_00_) | 4.86 | .62 | 7.87 |  | <.001 |
|  | Time (γ_10_) | 0.03 | .19 | 0.16 |  | .872 |
|  | Word Count (γ_20_) | -0.05 | .38 | -0.13 |  | .897 |
|  | Condition (γ_01_) | 0.06 | .11 | 0.54 |  | .587 |
|  | Trait Open (γ_02_) | 1.72 | .92 | 1.87 |  | .063 |
|  | Trait Open*Condition (γ_03_) | -0.34 | .54 | -0.63 |  | .529 |
|  | Trait Open*Time (γ_12_) | 0.06 | .12 | 0.52 |  | .601 |
|  | Time*Condition (γ_11_) | 0.06 | .12 | 0.54 |  | .587 |
|  | Time*Word Count (γ_30_) | 0.00 | .00 | 3.75 |  | <.001 |
|  | Condition*Word Count(γ_21_) | -0.00 | .00 | -1.19 |  | .234 |
|  | Intercept (τ_00_) | 2.32 | .32 |  | 7.35 | <.001 |
|  | Residual (σ^2^) | 2.66 | .17 |  | 15.75 | <.001 |
|  |  |  |  |  |  |  |
| E | Intercept (γ_00_) | 4.65 | .42 | 10.96 |  | <.001 |
|  | Time (γ_10_) | 0.13 | .06 | 2.25 |  | .025 |
|  | Word Count (γ_20_) | -0.00 | .00 | -1.31 |  | .191 |
|  | Condition (γ_01_) | 0.09 | .25 | 0.36 |  | .718 |
|  | Trait Open (γ_02_) | 1.36 | .27 | 5.03 |  | <.001 |
|  | Time*Word Count (γ_30_) | 0.00 | .00 | 3.94 |  | <.001 |
|  | Intercept (τ_00_) | 2.29 | .31 |  | 7.38 | <.001 |
|  | Residual (σ^2^) | 2.66 | .17 |  | 15.82 | <.001 |
|  |  |  |  |  |  |  |

Figure 7. MLM models for alternate uses task ‘flexibility’ scores (daily assessments).

|  | Openness | | Control | |  |
| --- | --- | --- | --- | --- | --- |
|  | *M* | *SD* | *M* | *SD* | *d* |
| Day 1 (Brick) | 7.72 | 2.97 | 7.57 | 3.82 | .04 |
| Day 2 (Newspaper) | 8.37 | 3.20 | 8.56 | 3.86 | .05 |
| Day 3 (Paperclip) | 6.56 | 3.22 | 6.67 | 3.28 | .03 |
| Day 4 (Pillow) | 6.80 | 3.15 | 6.93 | 3.22 | .04 |
| Day 5 (Shoebox) | 6.81 | 3.16 | 6.82 | 3.20 | .00 |

Table 5. Alternate uses task ‘fluency’ descriptive statistics (daily assessments). Topics in the openness condition are aesthetics (task 1), emotions (task 2), ideas (task 3) and introspection (task 4); task 5 is the curiosity manipulation.

Figure 8. Mean alternate uses task ‘fluency’ scores over time (daily assessments). Topics in the openness condition are aesthetics (task 1), emotions (task 2), ideas (task 3) and introspection (task 4); task 5 is the curiosity manipulation.

| Model |  | Parameter Estimates | Standard Error | *t* | *z* | *p* |
| --- | --- | --- | --- | --- | --- | --- |
|  |  |  |  |  |  |  |
| A | Intercept (γ_00_) | 8.12 | .24 | 33.23 |  | <.001 |
|  | Time (γ_10_) | -0.33 | .05 | -6.50 |  | <.001 |
|  | Intercept (τ_00_) | 6.95 | .79 | 8.83 |  | <.001 |
|  | Residual (σ^2^) | 4.33 | .24 | 18.40 |  | <.001 |
|  |  |  |  |  |  |  |
| B | Intercept (γ_00_) | 8.11 | .27 | 30.50 |  | <.001 |
|  | Time (γ_10_) | -0.32 | .05 | -5.99 |  | <.001 |
|  | Intercept (τ_00_) | 9.43 | 1.48 |  | 6.36 | <.001 |
|  | Time (τ_11_) | 0.09 | .06 |  | 1.41 | .079 |
|  | Cov (τ_10_) | -0.54 | .26 |  | -2.09 | .036 |
|  | Residual (σ^2^) | 4.11 | .26 |  | 15.83 | <.001 |
|  |  |  |  |  |  |  |
| C | Intercept (γ_00_) | 8.00 | .79 | 10.11 |  | <.001 |
|  | Time (γ_10_) | -029 | .16 | -1.79 |  | .074 |
|  | Condition (γ_01_) | 0.08 | .49 | 0.16 |  | .870 |
|  | Time*Condition (γ_11_) | -0.02 | .10 | -0.23 |  | .819 |
|  | Intercept (τ_00_) | 6.99 | .79 |  | 8.82 | <.001 |
|  | Residual (σ^2^) | 4.34 | .24 |  | 18.38 | <.001 |
|  |  |  |  |  |  |  |
| D | Intercept (γ_00_) | 7.85 | .87 | 9.07 |  | <.001 |
|  | Time (γ_10_) | -0.40 | .24 | -1.66 |  | .097 |
|  | Word Count (γ_20_) | 0.00 | .00 | 0.32 |  | .751 |
|  | Condition (γ_01_) | 0.21 | .53 | 0.40 |  | .692 |
|  | Trait Open (γ_02_) | 3.21 | 1.39 | 2.31 |  | .022 |
|  | Trait Open*Condition (γ_03_) | -0.80 | .84 | -0.96 |  | .340 |
|  | Trait Open*Time (γ_12_) | -0.14 | .16 | -0.86 |  | .390 |
|  | Time*Condition (γ_11_) | 0.03 | .15 | 0.18 |  | .859 |
|  | Time*Word Count (γ_30_) | 0.00 | .00 | 2.73 |  | .007 |
|  | Condition*Word Count(γ_21_) | -0.00 | .00 | -0.36 |  | .721 |
|  | Intercept (τ_00_) | 6.22 | .75 |  | 8.24 | <.001 |
|  | Residual (σ^2^) | 4.39 | .28 |  | 15.77 | <.001 |
|  |  |  |  |  |  |  |
| E | Intercept (γ_00_) | 7.79 | .65 | 12.07 |  | <.001 |
|  | Time (γ_10_) | -0.36 | .08 | -4.84 |  | <.001 |
|  | Word Count (γ_20_) | 0.00 | .00 | 0.20 |  | .843 |
|  | Condition (γ_01_) | 0.26 | .38 | 0.68 |  | .500 |
|  | Trait Open (γ_02_) | 1.67 | .42 | 3.97 |  | <.001 |
|  | Time*Word Count (γ_30_) | 0.00 | .00 | 2.61 |  | .009 |
|  | Intercept (τ_00_) | 6.19 | .75 |  | 8.30 | <.001 |
|  | Residual (σ^2^) | 4.38 | .28 |  | 15.84 | <.001 |
|  |  |  |  |  |  |  |

Table 6. MLM models for alternate uses task ‘fluency’ scores (daily assessments).

Figure 9. Probing time by word count interaction for alternate uses task fluency scores (daily assessments).

|  | Openness | | Control | |  |
| --- | --- | --- | --- | --- | --- |
|  | *M* | *SD* | *M* | *SD* | *d* |
| Day 1 (Brick) | 4.53 | 4.10 | 4.75 | 5.03 | .05 |
| Day 2 (Newspaper) | 4.65 | 3.90 | 5.30 | 5.11 | .14 |
| Day 3 (Paperclip) | 3.05 | 2.92 | 4.11 | 3.74 | .32 |
| Day 4 (Pillow) | 1.87 | 2.25 | 2.27 | 3.39 | .14 |
| Day 5 (Shoebox) | 1.19 | 2.11 | 1.12 | 2.20 | .03 |

Table 7. Alternate uses task ‘elaboration’ descriptive statistics (daily assessments). Topics in the openness condition are aesthetics (task 1), emotions (task 2), ideas (task 3) and introspection (task 4); task 5 is the curiosity manipulation.

Figure 10. Mean alternate uses task ‘elaboration’ scores over time (daily assessments). Topics in the openness condition are aesthetics (task 1), emotions (task 2), ideas (task 3) and introspection (task 4); task 5 is the curiosity manipulation.

| Model |  | Parameter Estimates | Standard Error | *t* | *z* | *p* |
| --- | --- | --- | --- | --- | --- | --- |
|  |  |  |  |  |  |  |
| A | Intercept (γ_00_) | 6.16 | .27 | 22.57 |  | <.001 |
|  | Time (γ_10_) | -0.99 | .06 | -15.79 |  | <.001 |
|  | Intercept (τ_00_) | 7.00 | .85 |  | 8.27 | <.001 |
|  | Residual (σ^2^) | 6.68 | .36 |  | 18.43 | <.001 |
|  |  |  |  |  |  |  |
| B | Intercept (γ_00_) | 6.08 | .37 | 16.40 |  | <.001 |
|  | Time (γ_10_) | -0.96 | .07 | -12.84 |  | <.001 |
|  | Intercept (τ_00_) | 21.61 | 2.78 |  | 7.76 | <.001 |
|  | Time (τ_11_) | 0.51 | .12 |  | 4.35 | <.001 |
|  | Cov (τ_10_) | -3.22 | .54 |  | -6.01 | <.001 |
|  | Residual (σ^2^) | 5.39 | .34 |  | 15.91 | <.001 |
|  |  |  |  |  |  |  |
| C | Intercept (γ_00_) | 5.03 | 1.20 | 4.21 |  | <.001 |
|  | Time (γ_10_) | -0.81 | .24 | -3.33 |  | <.001 |
|  | Condition (γ_01_) | 0.68 | .74 | 0.92 |  | .361 |
|  | Time*Condition (γ_11_) | -0.10 | .15 | -0.67 |  | .501 |
|  | Intercept (τ_00_) | 21.64 | 2.79 |  | 7.75 | <.001 |
|  | Time (τ_11_) | 0.51 | .12 |  | 4.36 | <.001 |
|  | Cov (τ_10_) | -3.23 | .54 |  | -6.00 | <.001 |
|  | Residual (σ^2^) |  |  |  |  |  |
|  |  |  |  |  |  |  |
| D | Intercept (γ_00_) | 4.91 | 1.30 | 3.78 |  | <.001 |
|  | Time (γ_10_) | -0.94 | .34 | -2.75 |  | .006 |
|  | Word Count (γ_20_) | 0.00 | .00 | 0.86 |  | .392 |
|  | Condition (γ_01_) | 0.50 | .80 | 0.63 |  | .530 |
|  | Trait Open (γ_02_) | 3.65 | 1.56 | 2.34 |  | .020 |
|  | Trait Open*Condition (γ_03_) | -0.93 | .85 | -1.09 |  | .276 |
|  | Trait Open*Time (γ_12_) | -0.17 | .23 | -0.76 |  | .450 |
|  | Time*Condition (γ_11_) | 0.09 | .21 | 0.41 |  | .684 |
|  | Time*Word Count (γ_30_) | 0.00 | .00 | 0.08 |  | .937 |
|  | Condition*Word Count(γ_21_) | -0.00 | .00 | -0.04 |  | .967 |
|  | Intercept (τ_00_) | 21.00 | 3.19 |  | 6.57 | <.001 |
|  | Time (τ_11_) | 0.64 | .23 |  | 2.76 | .003 |
|  | Cov (τ_10_) | -3.49 | .80 |  | -4.39 | <.001 |
|  | Residual (σ^2^) | 6.19 | .48 |  | 12.93 | <.001 |
|  |  |  |  |  |  |  |
| E | Intercept (γ_00_) | 4.51 | .72 | 6.25 |  | <.001 |
|  | Time (γ_10_) | -0.81 | .10 | -7.75 |  | <.001 |
|  | Word Count (γ_20_) | 0.00 | .00 | 3.09 |  | .002 |
|  | Condition (γ_01_) | 0.78 | .39 | 1.99 |  | .048 |
|  | Trait Open (γ_02_) | 1.68 | .42 | 3.94 |  | <.001 |
|  | Intercept (τ_00_) | 20.87 | 3.16 |  | 6.60 | <.001 |
|  | Time (τ_11_) | 0.62 | .23 |  | 2.72 | .003 |
|  | Cov (τ_10_) | -3.44 | .78 |  | -4.38 | <.001 |
|  | Residual (σ^2^) | 6.18 | .48 |  | 12.95 | <.001 |
|  |  |  |  |  |  |  |

Table 8. MLM models for alternate uses task ‘elaboration’ scores (daily assessments).

| Model E | Originality | Fluency | Elaboration | | Flexibility | |  |
| --- | --- | --- | --- | --- | --- | --- | --- |
| Intercept (γ_00_) | * | * | | * | | * | |
| Time (γ_10_) | * | * | | * | | * | |
| Condition (γ_01_) |  |  | | * | |  | |
| Trait Open | * | * | | * | | * | |
| Word Count (γ_20_) | * |  | | * | |  | |
| Time*Word Count (γ_12_) | N/A | * | | N/A | | * | |
| Intercept (τ_00_) | * | * | | * | | * | |
| Time (τ_11_) | N/A | N/A | | * | | N/A | |
| Cov (τ_10_) | N/A | N/A | | * | | N/A | |
| Residual (σ^2^) | * | * | | * | | * | |
| Inter Class Correlation (ICC) | .26 | .61 | | .51 | | .47 | |

Table 9. Summary of statistically significant findings from Model E for alternate uses task subscales. *p <.05. N/A ( Not Applicable) denotes models where the variable time was not allowed to randomly vary or an interaction was not retained in the model.

|  | Openness | | | Control | | |
| --- | --- | --- | --- | --- | --- | --- |
|  | n | *M* | *SD* | n | *M* | *SD* |
| Pretest | 66 | 14.45 | 5.44 | 70 | 13.96 | 4.23 |
| Posttest | 66 | 13.12 | 5.89 | 70 | 12.57 | 4.99 |

Table 10. Remote association task descriptive statistics (pretest and posttest).

Figure 11. Mean remote association task over time (pretest and posttest).

|  | Openness | | | Control | | |  |
| --- | --- | --- | --- | --- | --- | --- | --- |
|  | n | *M* | *SD* | n | *M* | *SD* | *d* |
| Pretest | 106 | 4.12 | .54 | 115 | 3.95 | .53 | .32 |
| Posttest | 86 | 4.11 | .57 | 93 | 4.14 | .57 | .05 |
| Follow up | 38 | 4.11 | .65 | 32 | 4.20 | .53 | .15 |

Table 11. Personal growth descriptive statistics (pretest, posttest, and follow up assessments).

Figure 12. Mean personal growth scores over time (pretest, posttest, and follow-up assessments).

| Model |  | Parameter Estimates | Standard Error | t | z | p |
| --- | --- | --- | --- | --- | --- | --- |
|  |  |  |  |  |  |  |
| A | Intercept (γ_00_) | 4.12 | 0.04 | 109.14 |  | <.001 |
|  | Time (γ_10_) | 0.05 | 0.02 | 2.69 |  | .008 |
|  | Intercept (τ_00_) | 0.19 | 0.02 |  | 7.76 | <.001 |
|  | Residual (σ^2^) | 0.07 | 0.01 |  | 10.16 | <.001 |
|  |  |  |  |  |  |  |
| B | Intercept (γ_00_) | 4.11 | 0.04 | 110.75 |  | <.001 |
|  | Time (γ_10_) | 0.06 | 0.02 | 2.61 |  | .010 |
|  | Intercept (τ_00_) | 0.19 | 0.03 |  | 6.84 | <.001 |
|  | Time (τ_11_) | 0.01 | 0.01 |  | 0.85 | .197 |
|  | Cov (τ_10_) | 0.00 | 0.01 |  | 0.10 | <.001 |
|  | Residual (σ^2^) | 0.06 | 0.01 |  | 6.58 | <.001 |
|  |  |  |  |  |  |  |
| C | Intercept (γ_00_) | 4.04 | 0.05 | 76.86 |  | <.001 |
|  | Time (γ_10_) | 0.09 | 0.03 | 2.88 |  | .004 |
|  | Condition (γ_01_) | 0.10 | 0.08 | 1.34 |  | .180 |
|  | Time*Condition (γ_11_) | -0.06 | 0.04 | -1.43 |  | .155 |
|  | Intercept (τ_00_) | 0.19 | 0.02 |  | 7.75 | <.001 |
|  | Residual (σ^2^) | 0.07 | 0.01 |  | 10.15 | <.001 |
|  |  |  |  |  |  |  |
| D | Intercept (γ_00_) | 4.08 | 0.05 | 84.85 |  | <.001 |
|  | Time (γ_10_) | 0.08 | 0.03 | 2.79 |  | .006 |
|  | Word Count (γ_20_) | 0.00 | 0.00 | 1.12 |  | .266 |
|  | Condition (γ_01_) | 0.06 | 0.07 | 0.83 |  | .406 |
|  | Trait Open (γ_02_) | 0.21 | 0.10 | 2.04 |  | .043 |
|  | Trait Open*Condition (γ_03_) | 0.29 | 0.14 | 2.07 |  | .040 |
|  | Trait Open*Time (γ_12_) | 0.02 | 0.04 | 0.48 |  | .633 |
|  | Time*Condition (γ_11_) | -0.06 | 0.04 | -1.45 |  | .149 |
|  | Time*Word Count (γ_30_) | 0.00 | 0.00 | -.05 |  | .958 |
|  | Condition*WordCount(γ_21_) | 0.00 | 0.00 | 1.28 |  | .202 |
|  | Intercept (τ_00_) | 0.14 | 0.02 |  | 7.24 | <.001 |
|  | Residual (σ^2^) | 0.07 | 0.01 |  | 10.11 | <.001 |
|  |  |  |  |  |  |  |
| E | Intercept (γ_00_) | 4.10 | .05 | 88.40 |  | <.001 |
|  | Time (γ_10_) | 0.05 | .02 | 2.60 |  | .010 |
|  | Word Count (γ_20_) | 0.00 | .00 | 2.38 |  | .018 |
|  | Condition (γ_01_) | 0.03 | .06 | 0.39 |  | .695 |
|  | Trait Open (γ_02_) | 0.21 | .10 | 2.09 |  | .038 |
|  | Trait Open*Condition (γ_03_) | 0.33 | .14 | 2.44 |  | .016 |
|  | Intercept (τ_00_) | 0.14 | .02 |  | 7.31 | <.001 |
|  | Residual (σ^2^) | 0.07 | .01 |  | 10.19 | <.001 |
|  |  |  |  |  |  |  |

Table 12. MLM models for personal growth (pretest, posttest, and follow-up assessments).

Figure 13. Simple slopes for condition by trait openness interaction predicting personal growth (pretest, posttest, and follow-up assessments).

|  | Openness | | | Control | | |  |
| --- | --- | --- | --- | --- | --- | --- | --- |
|  | n | *M* | *SD* | n | *M* | *SD* | *d* |
| Daily Task 1 | 96 | 5.53 | 1.18 | 107 | 5.70 | 1.16 | .15 |
| Daily Task 2 | 99 | 5.76 | 1.35 | 104 | 5.54 | 1.31 | .17 |
| Daily Task 3 | 94 | 5.67 | 1.31 | 99 | 5.74 | 1.35 | .05 |
| Daily Task 4 | 89 | 5.79 | 1.26 | 93 | 5.66 | 1.47 | .09 |
| Daily Task 5 | 88 | 5.89 | 1.22 | 97 | 6.02 | 1.23 | .11 |
| Overall | 466 | 5.73 | 1.27 | 500 | 5.72 | 1.31 | .01 |

Table 13. Authenticity descriptive statistics (daily assessments). Topics in the openness condition are aesthetics (task1), emotions (task 2), ideas (task3) and introspection (task 4); task 5 is the curiosity manipulation.

Figure 14. Mean authenticity scores over time (daily assessments). Topics in the openness condition are aesthetics (task 1), emotions (task 2), ideas (task 3) and introspection (task 4); task 5 is the curiosity manipulation.

| Model |  | Parameter Estimates | Standard Error | *t* | *z* | *p* |
| --- | --- | --- | --- | --- | --- | --- |
|  |  |  |  |  |  |  |
| A | Intercept (γ_00_) | 5.49 | 0.09 | 59.85 |  | <.001 |
|  | Time (γ_10_) | 0.07 | 0.02 | 3.91 |  | <.001 |
|  | Intercept (τ_00_) | 1.03 | 0.12 |  | 8.94 | <.001 |
|  | Residual (σ^2^) | 0.66 | 0.03 |  | 19.27 | <.001 |
|  |  |  |  |  |  |  |
| B | Intercept (γ_00_) | 5.49 | 0.09 | 60.39 |  | <.001 |
|  | Time (γ_10_) | 0.07 | 0.02 | 3.25 |  | .001 |
|  | Intercept (τ_00_) | 1.08 | 0.18 |  | 5.90 | <.001 |
|  | Time (τ_11_) | 0.03 | 0.01 |  | 2.72 | .003 |
|  | Cov (τ_10_) | -0.05 | 0.04 |  | -1.28 | .200 |
|  | Residual (σ^2^) | 0.59 | 0.04 |  | 16.18 | <.001 |
|  |  |  |  |  |  |  |
| C | Intercept (γ_00_) | 5.48 | 0.13 | 43.35 |  | <.001 |
|  | Time (γ_10_) | 0.07 | 0.03 | 2.45 |  | .015 |
|  | Condition (γ_01_) | 0.03 | 0.18 | 0.19 |  | .850 |
|  | Time*Condition (γ_11_) | -0.01 | 0.04 | -0.17 |  | .868 |
|  | Intercept (τ_00_) | 1.09 | 0.18 |  | 5.91 | <.001 |
|  | Time (τ_11_) | 0.03 | 0.01 |  | 2.75 | .003 |
|  | Cov (τ_10_) | -0.05 | 0.04 |  | -1.31 | .190 |
|  | Residual (σ^2^) | 0.59 | 0.04 |  | 16.18 | <.001 |
|  |  |  |  |  |  |  |
| D | Intercept (γ_00_) | 5.59 | 0.14 | 42.68 |  | <.001 |
|  | Time (γ_10_) | 0.01 | 0.05 | 0.27 |  | .791 |
|  | Condition (γ_01_) | -0.14 | 0.19 | -0.75 |  | .454 |
|  | Word Count (γ_20_) | 0.00 | 0.00 | 1.00 |  | .319 |
|  | Trait Open (γ_02_) | -0.02 | 0.26 | -0.09 |  | .925 |
|  | Time*Trait Open(γ_12_) | -0.07 | 0.07 | -1.03 |  | .305 |
|  | Trait Open*Condition (γ_03_) | 1.21 | 0.32 | 3.79 |  | <.001 |
|  | Time*Condition (γ_11_) | 0.07 | 0.07 | 1.05 |  | .295 |
|  | Time*Word Count (γ_12_) | 0.00 | 0.00 | 0.30 |  | .763 |
|  | Condition*Word Count (γ_21_) | 0.00 | 0.00 | 0.05 |  | .961 |
|  | Intercept (τ_00_) | 0.91 | 0.19 |  | 4.88 | <.001 |
|  | Time (τ_11_) | 0.09 | 0.02 |  | 3.95 | <.001 |
|  | Cov (τ_10_) | -0.07 | 0.05 |  | -1.37 | .017 |
|  | Residual (σ^2^) | 0.50 | 0.04 |  | 13.14 | <.001 |
|  |  |  |  |  |  |  |
| E | Intercept (γ_00_) | 5.49 | .24 | 23.12 |  | <.001 |
|  | Time (γ_10_) | 0.05 | .03 | 1.40 |  | .161 |
|  | Condition (γ_01_) | 0.02 | .14 | 0.13 |  | .896 |
|  | Word Count (γ_20_) | 0.00 | .00 | 4.03 |  | <.001 |
|  | Trait Open (γ_02_) | 2.27 | .50 | 4.52 |  | <.001 |
|  | Trait Open*Condition(γ_03_) | -1.21 | .32 | -3.80 |  | <.001 |
|  | Intercept (τ_00_) | 0.90 | .18 |  | 4.89 | <.001 |
|  | Time (τ_11_) | 0.09 | .04 |  | 3.95 | <.001 |
|  | Cov (τ_10_) | -0.07 | .05 |  | -1.31 | .190 |
|  | Residual (σ^2^) | 0.50 | .04 |  | 13.20 | <.001 |
|  |  |  |  |  |  |  |

Table 14 MLM models for authenticity (daily assessments).

Figure 15. Simple slopes for condition by trait openness interaction predicting authenticity scores (daily assessments).

|  | Openness | | | Control | | |  |
| --- | --- | --- | --- | --- | --- | --- | --- |
|  | n | *M* | *SD* | n | *M* | *SD* | *d* |
| Pretest | 106 | 4.76 | 1.02 | 115 | 4.67 | 1.12 | .08 |
| Posttest | 84 | 5.16 | 1.07 | 92 | 5.24 | 1.16 | .07 |
| Follow up | 38 | 5.13 | 1.11 | 32 | 5.09 | 1.08 | .03 |

Table 15. Authenticity descriptive statistics (pretest, posttest, and follow up assessments).

Figure 16. Mean authenticity scores over time (pretest, posttest, and follow up assessments).

| Model |  | Parameter Estimates | Standard Error | *t* | *z* | *p* |
| --- | --- | --- | --- | --- | --- | --- |
|  |  |  |  |  |  |  |
| A | Intercept (γ_00_) | 5.47 | 0.17 | 30.67 |  | <.001 |
|  | Time (γ_10_) | -0.18 | 0.13 | -1.39 |  | .170 |
|  | Intercept (τ_00_) | 0.72 | 0.15 |  | 4.76 | <.001 |
|  | Residual (σ^2^) | 0.52 | 0.10 |  | 5.19 | <.001 |
|  |  |  |  |  |  |  |
| B | Intercept (γ_00_) | 5.47 | 0.18 | 30.72 |  | <.001 |
|  | Time (γ_10_) | -0.18 | 0.13 | -1.40 |  | .168 |
|  | Intercept (τ_00_) | 0.69 | 0.52 |  | 1.32 | .094 |
|  | Time (τ_11_) | 0.00 | 0.20 |  | 0.00 | .500 |
|  | Cov (τ_10_) | 0.01 | 0.32 |  | 0.05 | .963 |
|  | Residual (σ^2^) |  |  |  |  |  |
|  |  |  |  |  |  |  |
| C | Intercept (γ_00_) | 5.63 | 0.25 | 22.12 |  | <.001 |
|  | Time (γ_10_) | -0.30 | 0.19 | -1.61 |  | .114 |
|  | Condition (γ_01_) | -0.32 | 0.36 | -0.88 |  | .378 |
|  | Time*Condition (γ_11_) | 0.23 | 0.25 | 0.90 |  | .372 |
|  | Intercept (τ_00_) | 0.74 | 0.15 |  | 4.79 | <.001 |
|  | Residual (σ^2^) | 0.52 | 0.10 |  | 5.15 | .001 |
|  |  |  |  |  |  |  |
| D | Intercept (γ_00_) | 5.60 | 0.27 | 21.03 |  | <.001 |
|  | Time (γ_10_) | -0.28 | 0.20 | -1.39 |  | .171 |
|  | Word Count (γ_20_) | 0.00 | 0.00 | 0.42 |  | .678 |
|  | Condition (γ_01_) | -0.34 | 0.36 | -0.94 |  | .349 |
|  | Trait Open (γ_02_) | -0.44 | 0.41 | -1.08 |  | .282 |
|  | Trait Open*Condition (γ_03_) | 0.56 | 0.37 | 1.52 |  | .132 |
|  | Trait Open*Time (γ_12_) | 0.08 | 0.27 | 0.30 |  | .768 |
|  | Time*Condition (γ_11_) | 0.19 | 0.26 | 0.73 |  | .472 |
|  | Time*Word Count (γ_30_) | 0.00 | 0.00 | -0.27 |  | .785 |
|  | Condition*Word Count (γ_21_) | 0.00 | 0.00 | 1.87 |  | .064 |
|  | Intercept (τ_00_) | 0.68 | 0.15 |  | 4.51 | <.001 |
|  | Residual (σ^2^) | 0.53 | 0.10 |  | 5.12 | <.001 |
|  |  |  |  |  |  |  |
| E | Intercept (γ_00_) | 5.50 | .19 | 28.24 |  | <.001 |
|  | Time (γ_10_) | -0.19 | .13 | -1.49 |  | .147 |
|  | Word Count (γ_20_) | 0.00 | .00 | 0.04 |  | .968 |
|  | Condition (γ_01_) | -0.19 | .17 | -0.71 |  | .480 |
|  | Trait Open (γ_02_) | -0.06 | .18 | -0.33 |  | .743 |
|  | Condition*Word Count (γ_21_) | 0.00 | .00 | 2.47 |  | .015 |
|  | Intercept (τ_00_) | 0.69 | .15 |  | 4.61 | <.001 |
|  | Residual (σ^2^) | 0.52 | .10 |  | 5.21 | <.001 |
|  |  |  |  |  |  |  |

Table 16. MLM models for authenticity (pretest, posttest and follow-up assessments).

Figure 17. Simple slopes for condition by trait openness interaction predicting authenticity (posttest, follow-up assessments).

|  | Daily Logs | | | | | Pretest, Posttest, Follow Up | | | | | | |
| --- | --- | --- | --- | --- | --- | --- | --- | --- | --- | --- | --- | --- |
| Model E | Authenticity | Effort | PA | NA |  | Authenticity | Effort | PA | | NA | | Personal Growth |
| Intercept (γ_00_) | * | * | * | * |  | * | * | * | * | | * | |
| Time (γ_10_) |  | * | * | * |  |  | * | * | * | | * | |
| Condition (γ_01_) |  |  | * |  |  |  |  |  |  | |  | |
| Word Count (γ_20_) | * | * | * | * |  |  |  |  |  | | * | |
| Trait Open | * | * | * |  |  |  |  |  | † | | * | |
| Time*Trait Open | - | - | - | - |  | - | - | - | - | | - | |
| Trait Open*Condition | * | * | * | - |  | - | - | * | - | | * | |
| Time*Condition (γ_11_) | - | - | - | † |  | - | - |  | - | | - | |
| Time*Word Count (γ_12_) | - | - | - | - |  | - | - | - | - | | - | |
| Condition*Word Count (γ_21_) | - | * | - | * |  | * | * | - | - | | - | |
| Time*Time | - | - | - | - |  | - | * | * | * | | - | |
| Time*Time*Condition | - | - | - | - |  | - |  |  |  | | - | |
| Intercept (τ_00_) | * | * | * | * |  | * | * | * | * | | * | |
| Time (τ_11_) | * | * | * | * |  | - | - | * | - | | - | |
| Cov (τ_10_) |  |  |  |  |  | - | - |  | - | | - | |
| Residual (σ^2^) | * | * | * | * |  | * | * | * | * | | * | |
| Inter Class Correlation (ICC) | .61 | .62 | .59 | .71 |  | .58 |  | .54 | .60 | | .73 | |

Table 17. Summary of statistically significant findings from Model E across all tests.*p <.05, †p = .08. Dashes (“ -“) or denote models where the variable was not included in the model.

|  | Openness | | | Control | | |  |
| --- | --- | --- | --- | --- | --- | --- | --- |
|  | n | *M* | *SD* | n | *M* | *SD* | *d* |
| Daily Task 1 | 96 | 2.86 | .80 | 107 | 2.64 | .87 | .26 |
| Daily Task 2 | 99 | 2.77 | .95 | 104 | 2.48 | .93 | .31 |
| Daily Task 3 | 94 | 2.70 | .83 | 99 | 2.38 | .95 | .36 |
| Daily Task 4 | 89 | 2.52 | .91 | 93 | 2.28 | .88 | .27 |
| Daily Task 5 | 88 | 2.60 | .86 | 97 | 2.57 | .82 | .04 |
| Overall | 466 | 2.69 | .88 | 500 | 2.48 | .90 | .24 |

Table 18 Positive affect descriptive statistics (daily assessments). Topics in the openness condition are aesthetics (task 1), emotions (task 2), ideas (task 3) and introspection (task 4); task 5 is the curiosity manipulation.

Figure 18. Mean positive affect scores across time (daily assessments). Topics in the openness condition are aesthetics (task 1), emotions (task 2), ideas (task 3) and introspection (task 4); task 5 is the curiosity manipulation.

| Model |  | Parameter Estimates | Standard Error | *t* | *z* | *p* |
| --- | --- | --- | --- | --- | --- | --- |
|  |  |  |  |  |  |  |
| A | Intercept (γ_00_) | 2.76 | 0.06 | 43.66 |  | <.001 |
|  | Time (γ_10_) | -0.06 | 0.01 | -4.78 |  | <.001 |
|  | Intercept (τ_00_) | 0.47 | 0.05 |  | 8.86 | <.001 |
|  | Residual (σ^2^) | 0.33 | 0.02 |  | 19.28 | <.001 |
|  |  |  |  |  |  |  |
| B | Intercept (γ_00_) | 276 | 0.07 | 40.25 |  | <.001 |
|  | Time (γ_10_) | -0.06 | 0.02 | -4.19 |  | <.001 |
|  | Intercept (τ_00_) | 0.67 | 0.10 |  | 6.58 | <.001 |
|  | Time (τ_11_) | 0.02 | 0.01 |  | 3.03 | .001 |
|  | Cov (τ_10_) | -0.05 | 0.02 |  | -2.80 | .005 |
|  | Residual (σ^2^) | 0.29 | 0.02 |  | 16.60 | <.001 |
|  |  |  |  |  |  |  |
| C | Intercept (γ_00_) | 2.62 | 0.09 | 27.77 |  | <.001 |
|  | Time (γ_10_) | -0.04 | 0.02 | -2.09 |  | .037 |
|  | Condition (γ_01_) | 0.31 | 0.14 | 2.29 |  | .023 |
|  | Time*Condition (γ_11_) | -0.04 | 0.03 | -1.36 |  | .175 |
|  | Intercept (τ_00_) | 0.64 | 0.10 |  | 648 | <.001 |
|  | Time (τ_11_) | 0.02 | 0.01 |  | 2.99 | .001 |
|  | Cov (τ_10_) | -0.05 | 0.02 |  | -2.69 | .007 |
|  | Residual (σ^2^) | 0.29 | 0.02 |  | 16.59 | <.001 |
|  |  |  |  |  |  |  |
| D | Intercept (γ_00_) | 2.69 | 0.09 | 28.82 |  | <.001 |
|  | Time (γ_10_) | -0.09 | 0.03 | -3.37 |  | <.001 |
|  | Condition (γ_01_) | 0.27 | 0.13 | 2.02 |  | .045 |
|  | Word Count (γ_20_) | 0.00 | 0.00 | 1.03 |  | .305 |
|  | Trait Open(γ_02_) | -0.37 | 0.18 | -2.01 |  | .045 |
|  | Time*Trait Open (γ_12_) | 0.03 | 0.04 | 0.67 |  | .501 |
|  | Condition*Trait Open(γ_03_) | 0.79 | 0.23 | 3.50 |  | <.001 |
|  | Time*Condition (γ_11_) | -0.02 | 0.04 | -0.48 |  | .631 |
|  | Time*Word Count (γ_12_) | 0.00 | 0.00 | 1.00 |  | .319 |
|  | Condition*Word Count (γ_21_) | 0.00 | 0.00 | -1.74 |  | .082 |
|  | Intercept (τ_00_) | 0.47 | 0.09 |  | 4.96 | <.001 |
|  | Time (τ_11_) | 0.01 | 0.01 |  | 1.43 | .076 |
|  | Cov (τ_10_) | -0.01 | 0.02 |  | -0.32 | .748 |
|  | Residual (σ^2^) | 0.26 | 0.02 |  | 13.39 | <.001 |
|  |  |  |  |  |  |  |
| E | Intercept (γ_00_) | 3.16 | .17 | 18.68 |  | <.001 |
|  | Time (γ_10_) | -0.11 | .02 | -5.52 |  | <.001 |
|  | Condition (γ_01_) | -0.23 | .10 | -2.19 |  | .030 |
|  | Word Count (γ_20_) | 0.00 | .00 | 3.64 |  | <.001 |
|  | Trait Open (γ_02_) | 1.29 | .36 | 3.58 |  | <.001 |
|  | Condition*Trait Open (γ_03_) | -0.80 | .23 | -3.52 |  | <.001 |
|  | Intercept (τ_00_) | 0.45 | .09 |  | 4.89 | <.001 |
|  | Time (τ_11_) | 0.01 | .01 |  | 1.25 | .100 |
|  | Cov (τ_10_) | -0.00 | .02 |  | -0.12 | .906 |
|  | Residual (σ^2^) | 0.26 | .02 |  | 13.43 | <.001 |
|  |  |  |  |  |  |  |

Table 19. MLM models for positive affect (daily assessments).

Figure 19. Simple slopes for condition by trait openness interaction predicting positive affect (daily assessments).

|  | Openness | | | Control | | |  |
| --- | --- | --- | --- | --- | --- | --- | --- |
|  | n | M | SD | n | M | SD | *d* |
| Pretest | 106 | 3.18 | .64 | 115 | 3.21 | .88 | .04 |
| Posttest | 86 | 2.93 | .69 | 95 | 2.86 | .79 | .09 |
| Follow Up | 38 | 3.04 | .77 | 34 | 2.84 | .94 | .23 |

Table 20. Positive affect descriptive statistics (pretest, posttest and follow up assessments).

Figure 20.Mean positive affect scores across time (pretest, posttest, and follow up assessments).

| Model |  | Parameter Estimates | Standard Error | *t* | *z* | *p* |
| --- | --- | --- | --- | --- | --- | --- |
|  |  |  |  |  |  |  |
| A | Intercept (γ_00_) | 3.17 | 0.06 | 56.71 |  | <.001 |
|  | Time (γ_10_) | -0.18 | 0.04 | -4.70 |  | <.001 |
|  | Intercept (τ_00_) | 0.31 | 0.05 |  | 6.29 | <.001 |
|  | Residual (σ^2^) | 0.27 | 0.03 |  | 10.21 | <.001 |
|  |  |  |  |  |  |  |
| B | Intercept (γ_00_) | 3.17 | 0.05 | 57.96 |  | <.001 |
|  | Time (γ_10_) | -0.21 | 0.04 | -4.72 |  | <.001 |
|  | Intercept (τ_00_) | 0.35 | 0.07 |  | 5.39 | <.001 |
|  | Time (τ_11_) | 0.09 | 0.04 |  | 2.07 | .019 |
|  | Cov (τ_10_) | -0.04 | 0.04 |  | -1.01 | .313 |
|  | Residual (σ^2^) | 0.20 | 0.03 |  | 6.06 | <.001 |
|  |  |  |  |  |  |  |
| C | Intercept (γ_00_) | 3.20 | 0.08 | 41.57 |  | <.001 |
|  | Time (γ_10_) | -0.30 | 0.06 | -4.80 |  | <.001 |
|  | Condition (γ_01_) | -0.04 | 0.11 | -0.39 |  | .697 |
|  | Time*Condition (γ_11_) | 0.17 | 0.09 | 2.04 |  | .043 |
|  | Intercept (τ_00_) | 0.35 | 0.06 |  | 5.41 | <.001 |
|  | Time (τ_11_) | 0.09 | 0.04 |  | 1.95 | .025 |
|  | Cov (τ_10_) | -0.04 | 0.04 |  | -0.83 | .404 |
|  | Residual (σ^2^) | 0.20 | 0.03 |  | 6.11 | <.001 |
|  |  |  |  |  |  |  |
| D | Intercept (γ_00_) | 3.20 | 0.08 | 41.21 |  | <.001 |
|  | Time (γ_10_) | -0.30 | 0.06 | -4.81 |  | <.001 |
|  | Condition (γ_01_) | -0.05 | 0.11 | -0.45 |  | .656 |
|  | Word Count (γ_20_) | 0.00 | 0.00 | 0.56 |  | .578 |
|  | Trait Open(γ_02_) | -0.17 | 0.16 | -1.06 |  | .293 |
|  | Time*Trait Open(γ_02_) | 0.14 | 0.09 | 1.56 |  | .120 |
|  | Condition*Trait Open(γ_03_) | 0.43 | 0.22 | 1.96 |  | .052 |
|  | Time*Condition (γ_11_) | 0.17 | 0.09 | 1.99 |  | .048 |
|  | Time*Word Count (γ_12_) | 0.00 | 0.00 | 0.41 |  | .683 |
|  | Condition*Word Count (γ_21_) | 0.00 | 0.00 | -0.37 |  | .710 |
|  | Intercept (τ_00_) | 0.36 | 0.07 |  | 5.39 | <.001 |
|  | Time (τ_11_) | 0.08 | 0.04 |  | 1.91 | .028 |
|  | Cov (τ_10_) | -0.04 | 0.04 |  | -0.99 | .324 |
|  | Residual (σ^2^) | 0.197 | 0.03 |  | 6.11 | <.001 |
|  |  |  |  |  |  |  |
| E | Intercept (γ_00_) | 3.23 | 0.08 | 41.13 |  | <.001 |
|  | Time (γ_10_) | -0.60 | 0.14 | -4.42 |  | <.001 |
|  | Condition (γ_01_) | -0.06 | 0.11 | -0.55 |  | .585 |
|  | Word Count (γ_20_) | 0.00 | 0.00 | 0.60 |  | .552 |
|  | Trait Open(γ_02_) | -0.09 | 0.15 | -0.55 |  | .580 |
|  | Trait Open*Condition(γ_03_) | 0.41 | 0.21 | 1.95 |  | .053 |
|  | Time*Time(γ_40_) | 0.19 | 0.08 | 2.50 |  | .013 |
|  | Time*Time*Condition(γ_41_) | -0.04 | 0.11 | -0.33 |  | .745 |
|  | Intercept (τ_00_) | 0.37 | 0.07 |  | 5.62 | <.001 |
|  | Time (τ_11_) | 0.09 | 0.04 |  | 2.08 | .019 |
|  | Cov (τ_10_) | -0.05 | 0.04 |  | -1.18 | .236 |
|  | Residual (σ^2^) | 0.19 | 0.03 |  | 6.05 | <.001 |
|  |  |  |  |  |  |  |

Table 21. MLM models for positive affect (pretest, posttest, and follow-up assessments).

Figure 21. Simple slopes for condition by trait openness interaction predicting positive affect (pretest, posttest, and follow up assessments).

Table 22.

Summary of statistically significant findings from Model E across variables assessed during daily logs

|  | Pretest, Posttest, Follow Up | | | | |
| --- | --- | --- | --- | --- | --- |
| Model E | Authenticity | Effort | PA | NA | Personal Growth |
| Intercept (γ_00_) | 5.50 (.19)** | 3.58 (.12)** | 3.23 (.08)** | 2.44 (.07)** | 4.10 (.05)* |
| Time (γ_10_) | -0.19 (.13) | -0.98 (.18)** | -0.60 (.14)** | -0.74 (.09)** | 0.05 (.02)* |
| Condition (γ_01_) | -0.19 (.17) | 0.07 (.16) | -0.06 (.11) | -0.10 (.10) | 0.03 (.06) |
| Word Count (γ_20_) | 0.00 (.00) | -0.00 (.00) | 0.00 (.00) | -0.00 (.00) | 0.00 (.00)* |
| Trait Open(γ_02_) | -0.06 (.18) | 0.06 (.16) | -0.09 (.15) | 0.20 (.11) † | 0.21 (.10)* |
| Time*Trait Open | - | - | - | - | - |
| Trait Open*Condition | - | - | 0.41 (.21)* | - | 0.33 (.14)* |
| Time*Condition (γ_11_) | - | - | - | - | - |
| Time*Word Count (γ_12_) | - | - | - | - | - |
| Condition*Word Count (γ_21_) | 0.00 (.00)* | -0.00 (.00)* | - | - | - |
| Time*Time | - | 0.47 (.11)** | 0.19 (.08)* | 0.34 (.05)** | - |
| Time*Time*Condition | - | -0.03 (.07) | -0.04 (.11) | -0.04 (.03) | - |
| Intercept (τ_00_) | 0.69 (.15)** | 0.57 (.10)** | 0.37 (.07)** | 0.33 (.05)** | 0.14 (.02)** |
| Time (τ_11_) | - | - | 0.09 (.04)* | - | - |
| Cov (τ_10_) | - | - | -0.05 (.04) | - | - |
| Residual (σ^2^) | 0.52 (.10)** | 0.72 (.07)** | 0.19 (.03)** | 0.28 (.02)** | 0.07 (.01)** |
| Inter Class Correlation (ICC) | .58 | .82 | .54 | .60 | .73 |

*Note* .*p <.05, ** p<.001, †p = .06. Dashes (“ -“) denote instances where the variable was not included in the final model
